# Linear Peptides—A Combinatorial Innovation in the Venom of Some Modern Spiders

**DOI:** 10.3389/fmolb.2021.705141

**Published:** 2021-07-06

**Authors:** Lucia Kuhn-Nentwig, Heidi E. L. Lischer, Stano Pekár, Nicolas Langenegger, Maria J. Albo, Marco Isaia, Wolfgang Nentwig

**Affiliations:** ^1^ Institute of Ecology and Evolution, University of Bern, Bern, Switzerland; ^2^ Interfaculty Bioinformatics Unit, University of Bern, Bern, Switzerland; ^3^ Swiss Institute of Bioinformatics, Lausanne, Switzerland; ^4^ Department of Botany and Zoology, Faculty of Science, Masaryk University, Brno, Czech Republic; ^5^ Departamento de Ecología y Evolución, Facultad de Ciencias, UdelaR, Montevideo, Uruguay; ^6^ Departamento de Ecología y Biología Evolutiva, Instituto de Investigaciones Biologicas Clemente Estable, Montevideo, Uruguay; ^7^ Dipartimento di Scienze della Vita e Biologia dei Sistemi, University of Torino, Torino, Italy

**Keywords:** linear peptides, cytolytical peptides, complex precursors, NGS spider venom transcriptome analysis, venom protease, tachykinin-like peptides, lycosins, oxyopinins

## Abstract

In the venom of spiders, linear peptides (LPs), also called cytolytical or antimicrobial peptides, represent a largely neglected group of mostly membrane active substances that contribute in some spider species considerably to the killing power of spider venom. By next-generation sequencing venom gland transcriptome analysis, we investigated 48 spider species from 23 spider families and detected LPs in 20 species, belonging to five spider families (Ctenidae, Lycosidae, Oxyopidae, Pisauridae, and Zodariidae). The structural diversity is extraordinary high in some species: the lynx spider *Oxyopes heterophthalmus* contains 62 and the lycosid *Pardosa palustris* 60 different LPs. In total, we identified 524 linear peptide structures and some of them are in lycosids identical on amino acid level. LPs are mainly encoded in complex precursor structures in which, after the signal peptide and propeptide, 13 or more LPs (*Hogna radiata*) are connected by linkers. Besides *Cupiennius* species, also in Oxyopidae, posttranslational modifications of some precursor structures result in the formation of two-chain peptides. It is obvious that complex precursor structures represent a very suitable and fast method to produce a high number and a high diversity of bioactive LPs as economically as possible. At least in Lycosidae, Oxyopidae, and in the genus *Cupiennius*, LPs reach very high Transcripts Per Kilobase Million values, indicating functional importance within the envenomation process.

## Introduction

Spiders (Araneae) colonize nearly all terrestrial ecosystems and are with 49,400 confirmed species among the most successful invertebrate groups (WSC, 2021). They appeared at the end of the Carboniferous, some 315 million years ago. The oldest groups are mygalomorph spiders, while the modern araneomorph spiders came up with the Jurassic, some 200 million years ago (Selden and Penney, 2010). One of the most recent and most species-rich spider families, wolf spiders (Lycosidae) (Piacentini and Ramírez, 2019), evolved approximately 20 million years ago and belongs to a group of more than 30 families, the so-called retrolateral tibial apophysis clade (RTA-clade).

Most spiders are polyphagous and prey on arthropods, thus, a standard spider venom should be targeted towards a broad range of arthropods. Spider venom is therefore a rich source of low molecular mass compounds, enzymes, and proteins, and contains a high diversity of mainly cysteine containing neurotoxins (Kuhn-Nentwig et al., 2011a; Langenegger et al., 2019). The increasing availability of such a combinatorial library attracted more and more research with focus on medical applications (Saez and Herzig, 2019). A fourth group of major compounds are linear peptides (LPs) with membranolytic and further still unknown activities, also called antimicrobial peptides, but their occurrence among spiders is widely unknown. Up to now, the identification of LPs in spider venoms was limited to only eight spider species, all belonging to the mentioned RTA-clade, namely lycosids (Yan and Adams, 1998; Budnik et al., 2004; Melo-Braga et al., 2020), oxyopids (Corzo et al., 2002; Dubovskii et al., 2011), zodariids (Kozlov et al., 2006; Vassilevski et al., 2008; Dubovskii et al., 2015), ctenids (Pimenta et al., 2005), and the trechaleids *Cupiennius salei* and *C. getazi* (Kuhn-Nentwig et al., 2002; Kuhn-Nentwig, 2021).

Recently, the LP diversity has been investigated in depth in the venom gland transcriptome of the model spider *Cupiennius salei*, from which record-breaking 179 peptides were described. As expected, comparable LPs had also been identified in the venom gland transcriptome of the sister species *Cupiennius getazi* (Kuhn-Nentwig, 2021). This indicates a potentially rich source of combinatorial LPs, at least in this genus, and probably also in related taxa. It has already been argued that LPs represent a functionally very important venom component group, potentially at least as effective as neurotoxins (Kuzmenkov et al., 2016; Kuhn-Nentwig, 2021). By destroying unselectively negatively charged membranes in a target organism, LPs exert an own high insecticidal activity, but by doing so, they also support the effect of neurotoxins, thus giving neurotoxins free access to ion channels (Corzo et al., 2002; Wullschleger et al., 2005; Kuhn-Nentwig et al., 2019). Evolutionary speaking, however, it is also possible that LPs could become equal or more important than neurotoxins in the long run. This would imply that the LP strategy is faster, cheaper or more suitable to prevent resistance mechanisms than relying on the classical neurotoxins.

To consider such a strategy, it is important to know which spider taxa use LPs. So far, besides *Cupiennius*, LPs has only been known from four families, namely Ctenidae, Lycosidae, Oxyopidae, and Zodariidae, thus, it is unclear how general or widespread the development of LPs is. Traditionally, *Cupiennius* had been considered a member of Ctenidae, recently it was moved to Trechaleidae (Piacentini and Ramírez, 2019), but it had also been discussed as belonging to the Pisauridae or Lycosidae (for more details, see WSC 2021), thus, we keep it here separate.

NGS platforms, like IlluminaHiSeq 3,000, provide an opportunity for cost-efficient sequencing of many cDNA libraries, when avoiding the pitfall traps of possible transcriptome contaminations through this technology (Langenegger et al., 2019). To elucidate the occurrence of LPs in spider families, we analyzed NGS venom gland transcriptomes from 48 species belonging to 23 spider families, more or less related to the afore mentioned families with known LP structures and their wider relatives.

LPs of spiders are encoded in three different precursor structures (Kozlov et al., 2006). In simple precursors, after the signal peptide, the propeptide ends with a processing quadruplet motif (PQM), followed by a single peptide and a stop codon, whereas in binary precursors two peptides are connected to each other by a linker, and accordingly, in complex precursors three up to an unknown number (13 or more LPs) of peptides are connected. Linkers are short anionic peptides, N-terminally with an inverted PQM motif and C-terminally with a PQM motif, and they are likely to be excised by PQM proteases during maturation from precursor structures. The PQM motif is the specific recognition site for a specific venom protease, which releases the peptides during peptide maturation (Kozlov and Grishin, 2007; Langenegger et al., 2018; Kuhn-Nentwig, 2021).

We found LPs in 20 species, belonging to five spider families and *Cupiennius*, and a strikingly high structural diversity in Lycosidae. All these families are part of the RTA-clade, as noted above, where wolf spiders represent one of the most modern major spider family with nearly 2,500 species, corresponding to about 5% of all spider species (WSC, 2021). Our results support the idea that LPs are a remarkable innovation in spider venom, suitable to support or even replace the function of neurotoxins in an evolutionary context. These results on abundance and diversity of LPs so far characterized from *Cupiennius* and from five spider families opens the door into a surprising library of combinatorial peptides more or less unknown to science.

## Materials and Methods

### Spider Collection and cDNA Libraries of Venom Glands

Spiders were collected on public land and none of them are endangered or protected species. Some species were purchased on the pet market and about half of the species were kept or bred in the lab for a while until venom gland extraction. Spider identification was confirmed by experts when necessary. Spiders were anaesthetized with CO_2_ and venom was extracted once by electrical stimulation (3.5–7 V, 1–3 s, 1–3 times) until the venom glands were depleted. After electrical milking, venom glands were dissected in different time intervals (24, 48, 72 h, and 7 days) and stored in RNAlater (Qiagen) (Kuhn-Nentwig, 2021). An overview of 48 investigated spider species, the geographical origin and on transcriptomic sequencing is given in Supplementary Table S1.

cDNA libraries of spider venom glands were generated on an Illumina HiSeq 3,000 platform (University of Bern, Switzerland). Extraction of total RNA was performed combining phenol/chloroform extraction (in-house protocol) and the RNeasy mini kit (Qiagen). The assessment of RNA quantity and quality was done by Nanodrop, the Qubit RNA BR assay kit (Qubit 2.0 fluorometer; Thermo Fisher Scientific) and by an advanced analytical fragment analyzer system (fragment analyzer RNA kit, DNF-471, Agilent). cDNA library preparations were performed with the Illumina TruSeq-stranded mRNA prep kit using 1 µg of RNA for each library. Further sequencing was done on an Illumina HiSeq3000 sequencer using non-redundant double barcoding and selected fragments with lengths between 300 and 600 bp (Pippin HT system, Sage Science). All libraries were multiplexed (25% per lane) timely independent and with other non-arthropods, mostly genomic libraries of vertebrates, to diminish false positive identifications of LPs by index misassignment (Langenegger et al., 2019). The assembly of the resulting reads was done using Trinity version 2.1.1 or version 2.5.1 with default settings (Grabherr et al., 2011).

### Transcriptome Analysis of Illumina HiSeq3000 Data and Linear Peptide Identification

After assembly, the obtained contigs were translated into six reading frames. The translated sequences were blasted against an in-house database, composed of all spider LPs from UniprotKB/SwissProt, Arachnoserver and Venomzone (BLASTP, e-threshold 0.0001). Signal peptides were predicted using SignalP (SignalP v. 5.0) (Almagro Armenteros et al., 2019) and manually reviewed. Propeptides and potential linker sequences were manually annotated following the rules detailed in (Kuhn-Nentwig, 2021). All new identified cDNA sequences encoding possible LPs were used again as query and blasted against the spider transcriptomes using two different thresholds (BLASTP, e-threshold 0.01 and 0.0001).

Identified transcripts of each spider species were analyzed in terms of identification of signal peptides, propeptides, and peptides. If possible, overlapping amino acid sequences were used to identify possible N-terminal structures of transcript families (signal peptide, propeptide) or a possible C-terminus of a transcript family. Peptides and their N-terminal and C-terminal linkers were used to elongate the transcripts towards the signal peptide or the C-terminal end as described earlier (Kuhn-Nentwig, 2021) and were classified into different peptide families. New LPs were accepted as such, when the peptides exhibit N- and C-terminally at least a PQM or an iPQM motif (12 bps). Calculation of the TPM values (Transcripts Per Kilobase Million) of the transcriptomes was done according to (Wagner et al., 2012) using Kallisto version 0.44.0 (Bray et al., 2016). The characteristics of the deposited cDNA sequences (*n* = 600) are summarized in Supplementary Tables S2A–J.

### Evolutionary Analysis of Signal Peptides

Alignments of signal peptides were done by clustal omega (www.ebi.ac.uk; Madeira et al., 2019). The phylogenetic tree was estimated by a Maximum Likelihood method and JTT matrix-based model (Jones et al., 1992) using MEGA X (Kumar et al., 2018). The branch lengths correspond to the number of substitutions per site. The analysis was based on 140 signal peptides with lengths between 18 and 24 amino acid residues. However, all positions with less than 90% site coverage due to gaps and missing data were ignored (partial deletion option) and thus only 18 positions were in the final dataset.

To test the evolutionary hypothesis of LPs, we studied the relationship between the number of LPs in 48 species and the position of a particular family to which the investigated species belong to. The position was expressed as the number of nodes from the root of the phylogenetic tree (Wheeler et al., 2017). As the number of LPs were counts, we used generalized linear model (GLM) with the Poisson error structure (Pekár and Brabec, 2016). The analysis was performed within R environment (R_Core_Team, 2021).

The biochemical characterization of the peptides was done with an in-house protocol calculating molecular masses, isoelectric points (pIs), and contents of charged and hydrophobic amino acids. Peptide sequence logos were generated with WebLogo (Vers. 2.8.2) (Crooks et al., 2004). Peptide secondary structure prediction was calculated with the GOR method (Garnier et al., 1996). GraphPad PRISM Vers. 6.07 (GraphPad Software, San Diego, CA, United States) and Jalview Vers. 2.10.5 (Waterhouse et al., 2009) software was used for the visualization of results.

## Results

### Occurrence of Linear Peptides in Spider Venom Transcriptomes

**FIGURE 1:**
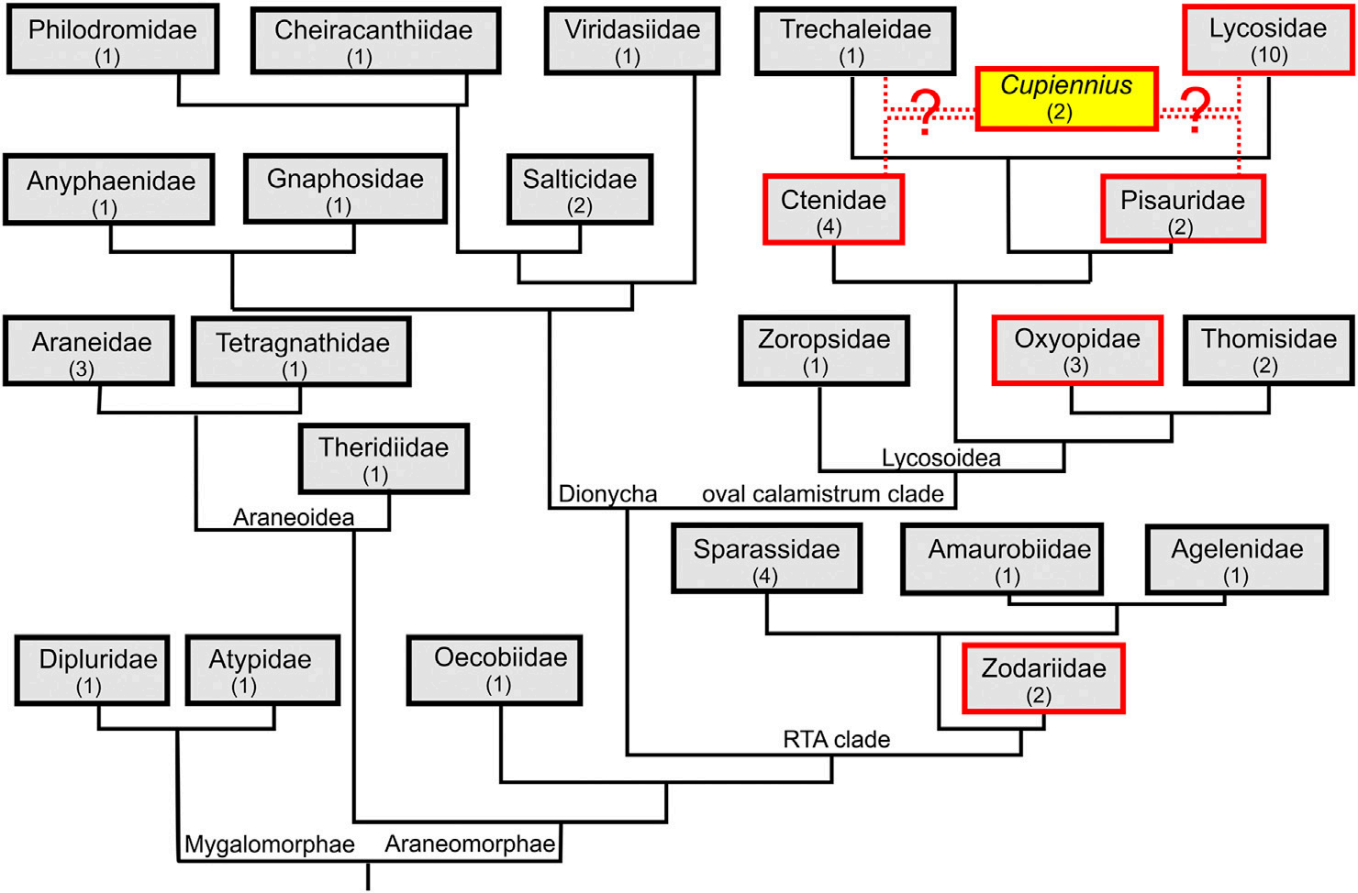
Truncated spider phylogeny with mapped presence of LPs. We investigated from all shown spider families the venom gland transcriptome of one or more spider species to identify linear peptide (LP) precursors. Families, in which LP precursors were identified are boxed in red and spider family transcriptomes without LP precursors are boxed in black. Numbers in brackets refer to the number of spider species per family. The phylogeny follows (Wheeler et al., 2017; Cheng and Piel, 2018; Fernández et al., 2018). The phylogenetic position of the genus *Cupiennius* is still under discussion.

We analyzed the venom gland transcriptomes of 48 spider species with two species belonging to mygalomorph spiders, five to the Araneoidea, one to the Oecobiidae at the basis of the RTA-clade, and 40 to the RTA-clade (Figure 1). No LP precursors have been identified in the transcriptomes of the mygalomorph spiders *Linothele megatheloides* and *Atypus piceus,* and in the araneomorph spider *Uroctea durandi*. Furthermore, in all species belonging to the Araneoidea (*Araneus angulatus*, *Larinioides sclopetarius*, *Nephila pilipes*, *Meta menardi, Latrodectus tredecimguttatus*), Oecobiidae, and those belonging to the Dionycha (*Anyphaena accentuata*, *Drassodes lapidosus*, *Viridasius fasciatus*, *Evarcha arcuata*, *Marpissa muscosa*, *Cheiracanthium* sp., and *Tibellus macellus*) no search results were obtained.

At the base of the RTA-clade, in zodariids, LP precursors had been reported for *Lachesana tarabaevi* (Kozlov et al., 2006). In two other species, *Zodarion cyrenaicum* and *Z. styliferum*, only a few, very peculiar precursors could be identified. No LPs were found in sparassids (*Eusparassus dufouri*, *Heteropoda venatoria*, *Isopeda villosa*), one amaurobiid (*Amaurobius ferox*), agelenid (*Eratigena atrica*), zoropsid (*Zoropsis spinimana*), and in thomisids (*Thomisus onustus*, *Xysticus cristatus*). Interestingly, in oxyopids, a neighbor family to the thomisids, in all three investigated species (*Oxyopes lineatus*, *O. heterophthalmus*, and *Peucetia striata*), a huge amount of different LP precursors was detected.

The most successful spiders in terms of numbers and variants of LP precursors within the Lycosoidea are *Cupiennius* species as recently published (Kuhn-Nentwig, 2021) and all so far included lycosids. All eleven investigated lycosids as *Hogna radiata* (from this species we included two populations from two different geographical areas: Spain and Italy), *Geolycosa vultuosa*, *Alopecosa cuneata*, *A. marikovskyi*, *Lycosa hispanica*, *L. praegrandis*, *Pardosa amentata*, *P. palustris*, *Trochosa ruricola*, and *Vesubia jugorum*, present a great number of structurally different LPs.

The ctenid *Phoneutria nigriventer* belongs to the best investigated spider species of South America concerning proteomics, transcriptomics, and neurophysiology (Diniz et al., 2018; Paiva et al., 2019). So far, the detection of tachykinin-like peptides in its venom (Pimenta et al., 2005) attracted our interests to investigate possible peptide precursors of such LPs. Surprisingly, in most investigated ctenids (*Phoneutria fera*, *Macroctenus kingsleyi*, and *Piloctenus haematostoma*), we discovered, besides the known tachykinin-like peptides, complex precursor structures encoding further so far unknown short LPs. *Ancylometes rufus*, from the same family, exhibits only one simple precursor which is more similar to oxyopids than to ctenids. Comparably, we identified in *Dolomedes okefinokensis* (Pisauridae) two LP precursors, but failed to detect any precursor structure in the other investigated pisaurid, *Pisaura mirabilis*. Quite recently, the genus *Cupiennius* was moved from Ctenidae to Trechaleidae (Piacentini and Ramírez, 2019), but we could not identify any LPs in the transcriptome of *Trechaleoides biocellata*.

**FIGURE 2:**
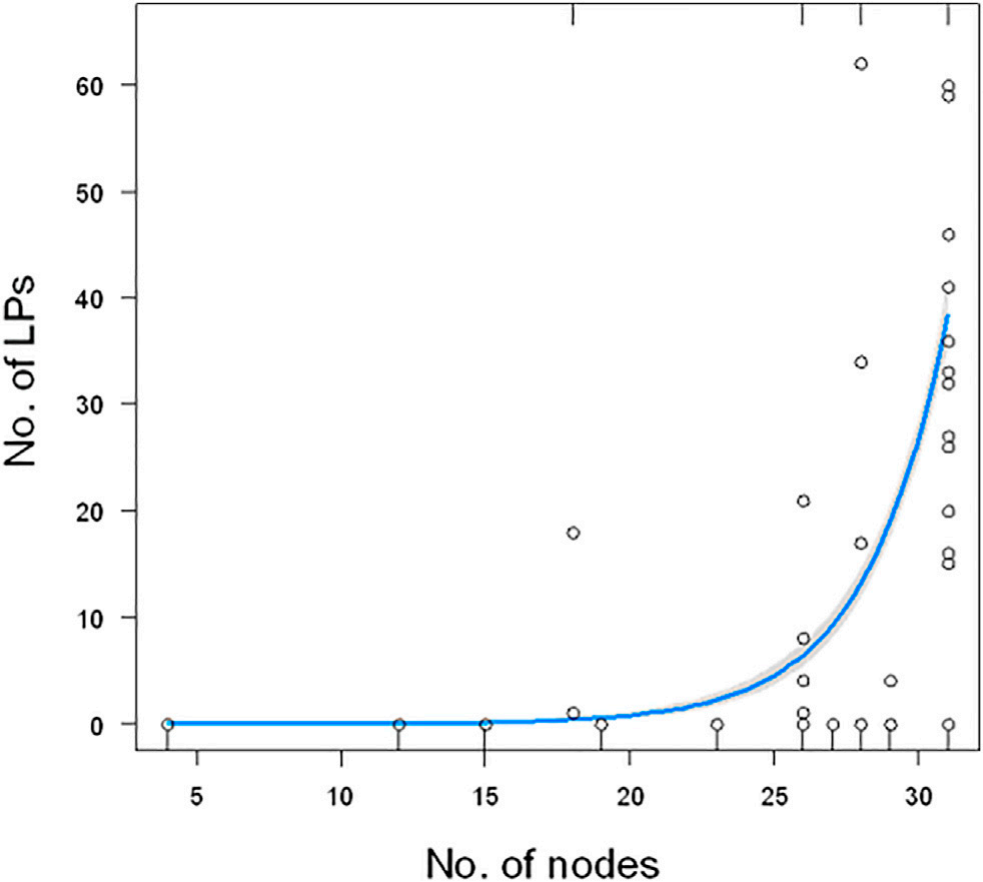
Relationship between the number of LPs and the number of nodes from the root of the tree. The plot does not show one extreme value (180 for *Cupiennius salei*). The blue line show the estimated model (GLM) with 95% confidence bands (gray).

We have studied the relationship between the number of detected LPs in 48 species (Supplementary Table S1) and the position of a particular family to which the investigated species belong to. The number of LPs was not similar among the study species. It significantly exponentially increased with the distance from the root of the phylogenetic tree (GLM, χ^2^_1_ = 328, *p* < 0.0001, Figure 2).

### Precursor Structures

**FIGURE 3:**
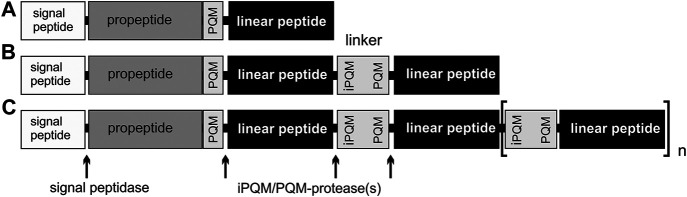
Overview of different precursor structures. **(A)** Simple precursor structure. **(B)** Binary precursor structure. **(C)** Complex precursor structure, which allows at least 13 connected peptides. Linkers are peptides, with an N-terminal iPQM motif and a C-terminal PQM motif, which connect linear peptides.

Precursors of LPs are composed of a signal peptide, followed by a propeptide region of different length with one or more C-terminal PQM motifs, and subsequent LPs, which are separated by linkers. These linkers are characterized N-terminally by an iPQM motif and C-terminally by a PQM motif. The PQM motif is composed of four amino acid residues, with an Arg residue at position −1 and at least one Glu residue at positions −2, −3, and/or −4, and the iPQM motif exhibits an Arg residue at position 1 and at least one Glu residue at position 2, 3, and/or 4. The precursors are divided into three types: simple and binary precursors which both encode only one or two peptides after the propeptides, and complex precursor structures, encoding more than two peptides. We found complex precursors giving rise to up to 13 mature LPs. Much higher number of peptides are possible, and they are always separated by linkers (Kuhn-Nentwig, 2021; Figure 3).

We analyzed 133 N-terminal precursors with complete signal peptide, propeptide and the first LP regions and we also took into account the ENA deposited transcripts of zodariids, oxyopids, and pisaurids. Interestingly, 35% (*n* = 46) of all precursors refer to simple precursors and 65% (*n* = 87) to binary and complex precursors. The total number of unique LPs per species that we obtained from different precursor structures is given in Table 1. From 812 identified LPs, 88.6% refer to complex precursors, 10.2% to simple and only 1.2% to binary precursor structures. About 46 individual signal peptides are responsible for the translocation of 83 individual simple transcripts, which can be explained due to minor mutations in the propeptides or LPs in simple precursors. Among complex and binary precursors, 729 individual LPs are encoded in only 87 transcripts families, thus on average, one complex precursor encodes about 8.4 LPs, and so far an identified maximum of 13 LPs.

**TABLE 1:** Overview of identified LPs deriving from simple, binary, and complex precursor structures from spider venom gland transcriptomes.

Spider family	Spider species	Analyzed N-terminal SP/PrP sequences	Individual peptides derived from			Nucleotide sequences deposited at ENA
			Simple precursors	Binary precursors	Complex precursors	
Lycosidae	*Alopecosa cuneata*	5			36	44
	*Alopecosa marikovskyi*	3			15	26
	*Geolycosa vultuosa*	4			46	49
	*Hogna radiata* (Spain)	3			33	44
	*Hogna radiata* (Italy)	3			27	31
	*Lycosa hispanica*	1			26	32
	*Lycosa praegrandis*	2			16	18
	*Pardosa amentata*	5°			32	35
	*Pardosa palustris*	5			60	69
	*Trochosa ruricola*	5			41	47
	*Vesubia jugorum*	2			20	22
Trechaleidae	*Cupiennius getazi*	5^§^	1		58^§^	51
	*Cupiennius salei*	9^§^	1		179^§^	238
Ctenidae	*Ancylometes rufus*	1	1			1
	*Macroctenus kingsleyi*	4			4	5
	*Phoneutria fera*	2			8	10
	*Piloctenus haematostoma*	3°			21	22
Oxyopidae	*Oxyopes heterophthalmus*	15°	12		50	59
	*Oxyopes takobius*	5^*^	4	2	3	^X^
	*Oxyopes lineatus*	11	9		25	34
	*Peucetia striata*	9°°	4		13	21
Pisauridae	*Dolomedes fimbriatus*	3^**^	5	2		^X^
	*Dolomedes okefinokensis*	3		4		8
Zodariidae	*Lachesana tarabaevi*	14^***^	27	2	6	^X^
	*Zodarion cyrenaicum*	1	1			1
	*Zodarion styliferum*	10	18			18
Sum of all		133	83	10	719	887

Combination of °two or °°three sequences for analysis of one N-terminal precursor structure [signal peptide (SP), propeptide (PrP), and the first peptide];

Precursors are from

* A0A5J6SIH8, A0A4D6Q2Y9, A0A5J6SEB1, A0A4D6Q7V4, F8J4S0.

** A0A0K1D8Z3, A0A0K1D8H4, A0A0K1D8X5.

*** Q1ELU5, Q1ELU4, Q1ELU1, P85253, Q1ELU3, C0HJV6, Q1ELT9, A0A1B3Z581, Q1ELU7, Q1ELU8, Q1ELV0, A0A1B3Z583, A0A1B3Z580, A0A1B3Z582.

^§^ Cupiennius salei and C. getazi: EMBL-EBI PRJEB42022.

x Investigated and deposited by others, not counted here (see text).

### Signal Peptides and Propeptides

Signal peptides are composed of 18–24 amino acid residues. Searching with BLASTP for further LPs in a new transcriptome was much more successful when using signal peptides together with the respective propeptides and LPs of known transcripts as query than using only LPs as query. The analysis of all signal peptides by the maximum likelihood method showed that they cluster mainly spider family and transcript family specific. Interestingly, besides genus specific transcript families, all lycosid spiders share one or two transcript families.

Obviously, for all spiders, the signal peptides of LP precursors are more related to each other than, within a spider species, signal peptides of LPs to signal peptides of neurotoxin precursors as shown for oxyopids (Supplementary Figure S1). Some of these neurotoxins (spiderines) are characterized by a cationic α-helical N-terminus and C-terminally an ICK motif (Vassilevski et al., 2013; Sachkova et al., 2014) or only an ICK motif (Corzo et al., 2002). The N-terminal α-helical structure of the spiderines is comparable to the cytolytically acting oxyopinin 1 and the cupiennin 1 and 2 families.

Propeptides are highly diverse in terms of length and may contain up to three iPQM/PQM motifs in its sequence (Kuhn-Nentwig, 2021), followed by a last PQM motif as cutting site before the first LP occurs. The most commonly identified C-terminal PQM motif among all analyzed propeptides (*n* = 133) was EEAR (38%), followed by XXER (X can be any amino acid residue in any position before Arg, 31%) and XEER (X can be any amino acid residue in any position before Arg, 27%). Importantly, in 4% of all PQM motifs, Glu is exchanged by Asp, XXDR (X can be any amino acid residue in any position before Arg, but not E). In general, propeptides are characterized by an acidic pI below 5 due to the increased presence of negatively charged Glu/Asp combined with a high content of hydrophobic amino acid residues. This charge distribution is often like a mirror image to the mainly positively charged Lys/Arg residues observed in many cytolytic peptides. Simple precursor structures were most frequently identified in zodariid spiders with lengths between 40 and 48 amino acid residues. In oxyopid spiders, we found short (27 amino acid residues) and long propeptides (50–57 amino acid residues) of simple precursors. Propeptides of complex precursors vary in length from ten (*T. ruricola* and *A. cuneata*) up to 86 amino acid residues (*O. lineatus*). More generally, propeptide sequences from lycosids are shorter than sequences from oxyopids. Propeptide sequences from *Cupiennius*, pisaurids, and ctenids are in a middle range (Supplementary Figure S2).

### Linkers

**FIGURE 4:**
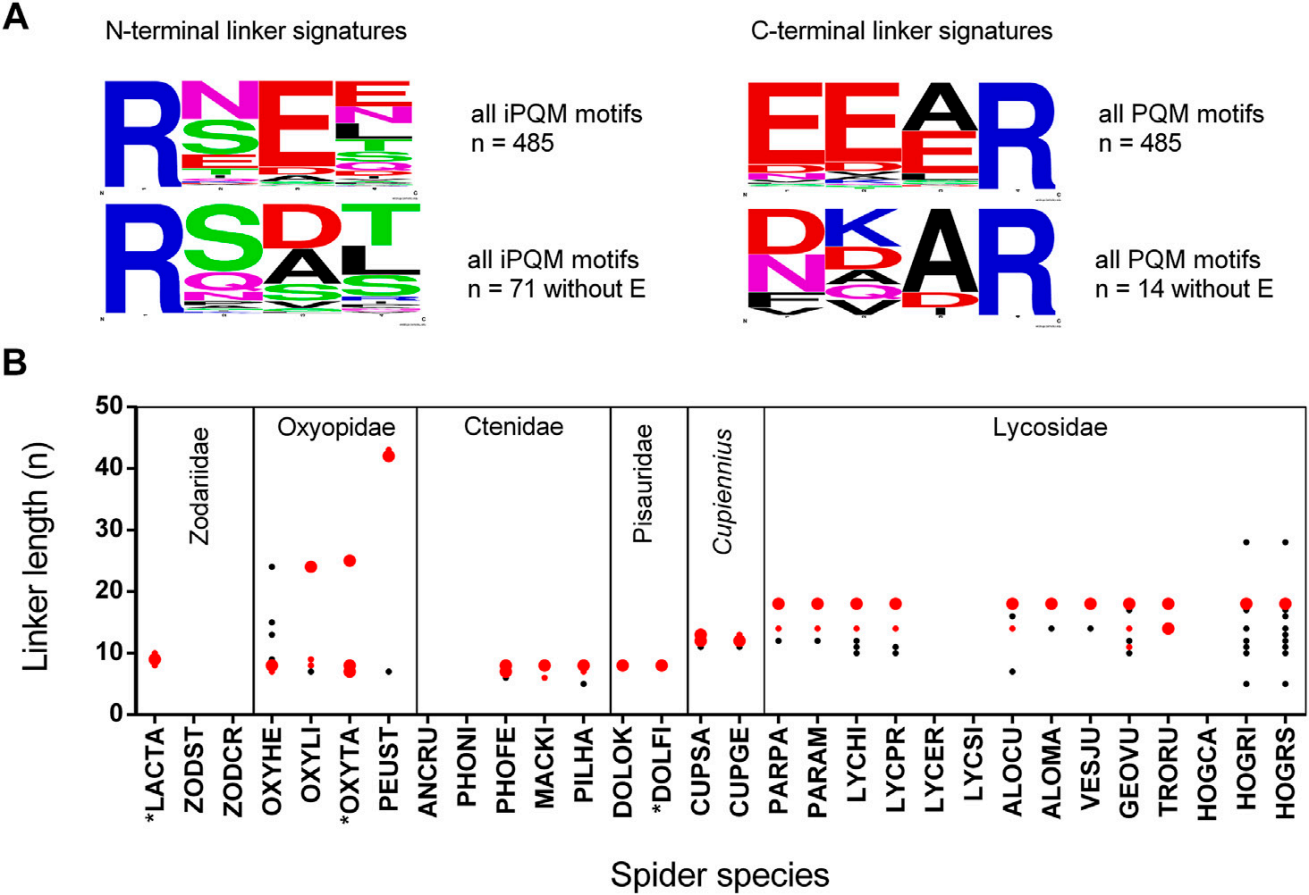
Signature, length, and abundance of linkers identified in binary/complex precursors of different spider families. **(A)** N-terminal (iPQM) and C-terminal (PQM) signatures of all identified linkers in the presence or absence of Glu. The relative frequency of each amino acid residue at a certain position of different N-termini and C-termini is given as a sequence logo. Cationic amino acids are given in blue, anionic amino acids in red, Asn and Gln in pink, polar amino acids in green, and hydrophobic amino acids in black. **(B)** Six spider families with the investigated species: The linker length is given as amino acid residues per peptide (n). The abundance of all linkers within a species is illustrated as big red dots (>30%), small red dots (>20%), and black small dots (<20%). *Data are from [A0A5J6SEB1, A0A5J6SEE5, A0A0K1D8Z3, Q1ELU5, Q1ELU4, Q1ELU8]. Species abbreviations are in alphabetical order: ALOCU, *Alopecosa radiata*; ALOMA, *Alopecosa marikovskyi*; ANCRU, *Ancylometes rufus*; CUPGE, *Cupiennius getazi*; CUPSA, *Cupiennius salei*; DOLFI, *Dolomedes fimbriatus*; DOLOK, *Dolomedes okefinokensis*; GEOVU, *Geolycosa vultuosa*; HOGCA, *Hogna carolinensis*; HOGRI, *Hogna radiata* (Italy); HOGRS, *Hogna radiata* (Spain); LACTA, *Lachesana tarabaevi*; LYCER, *Lycosa erythrognatha*; LYCHI, *Lycosa hispanica*; LYCPR, *Lycosa praegrandis*; LYCSI, *Lycosa singoriensis*; MACKI, *Macroctenus kingsleyi*; OXYHE, *Oxyopes heterophthalmus*; OXYLI, *Oxyopes lineatus*; OXYTA, *Oxypes takobius*; PARAM, *Pardosa amentata*; PARPA, *Pardosa palustris*; PEUST, *Peucetia striata*; PILHA, *Piloctenus haematostoma*; PHOFE, *Phoneutria fera*; PHONI, *Phoneutria nigriventer*; TRORU, *Trochosa ruricola*; VESJU, *Vesubia jugorum*; ZODCY, *Zodarion cyrenaicum*; ZODST, *Zodarion styliferum.*

Linkers are anionic peptides, which separate, and in doing so, connect different or identical LPs to each other within binary or complex precursors. As general rule, LPs within complex precursors are always separated by linkers showing N-terminally an iPQM motif and C-terminally a PQM motif. A linker starts N-terminally always with an Arg residue and defines with the following three amino acid residues the iPQM motif, and it ends C-terminally again with an Arg residue, terminating the PQM motif. We identified 485 unique linkers, and 14.6% of their iPQM motifs contain no Glu whereas only 2.9% of PQM motifs are missing a Glu residue. The iPQM motif seems to be more spider genus specific whereas the C-terminal PQM motif conforms to its definition with the occurrence of one to three Glu before the C-terminal Arg, and corresponds to the most often identified PQM motif EEAR of propeptides (Figure 4A; Supplementary Figure S3). Within different peptide precursors, linkers can be recurring or unique. Length and composition of the most abundant linkers per spider species is mainly genus specific as spider species of the same genus share some identical linkers. Interestingly, lycosid spiders also possess more individual linkers of different lengths as the other investigated genera. In several cases identical linkers have been identified in different genera of lycosid spiders. The shortest linkers were identified in *Piloctenus haematostoma* (ctenids) with RNEAR and in *Hogna radiata* (lycosids) with RSEER (Figure 4B). The last species, sampled in Italy (HOGRI) and Spain (HOGRS), and analyzed as two separated transcriptomes, is the only lycosid with an unusually long linker of 28 amino acid residues. This linker connects the first LP after the propeptide with the second one. Taking into account the extreme short propeptide of this transcript, it is possible that the first peptide was placed within the propeptide region. Furthermore, in oxyopids two extremely long linkers have been identified with 24/25 (*Oxyopes*) and 42/43 (*Peucetia*) amino acid residues, which separate LPs of different lengths. Likewise, the propeptides of such precursors are proportionally shorter than the propeptides in other peptide precursors of oxyopids. These precursors encode only variants of one LP family. Negligibly, less than 1% of all identified LPs show N- or C-terminal parts of linkers which are caused by indel mutations in the region of N-terminal or C-terminal Arg residues of the linkers and we found such cases only in lycosids.

### Linear Peptides

So far, the term LPs was used in the past mainly for short LPs without Cys residues in their sequences and a high cationic charge (Dubovskii et al., 2015). However, caused by the identification of two-chain peptides (CsTx-16) in complex precursors as single peptides within several short LPs, we added this peptide family to the overall LP family (Kuhn-Nentwig, 2021). Additionally, peptides exhibiting Rana-box-like motif containing two cysteines (Dubovskii et al., 2011), or such with one Cys, and other cationically charged long peptides (e.g. cytoinsectotoxins) (Vassilevski et al., 2008) were included in our analysis. Corresponding to previously published LPs, we named them here after the genus name, because identified peptides from different species of the same genus are often identical or very similar.

**FIGURE 5:**
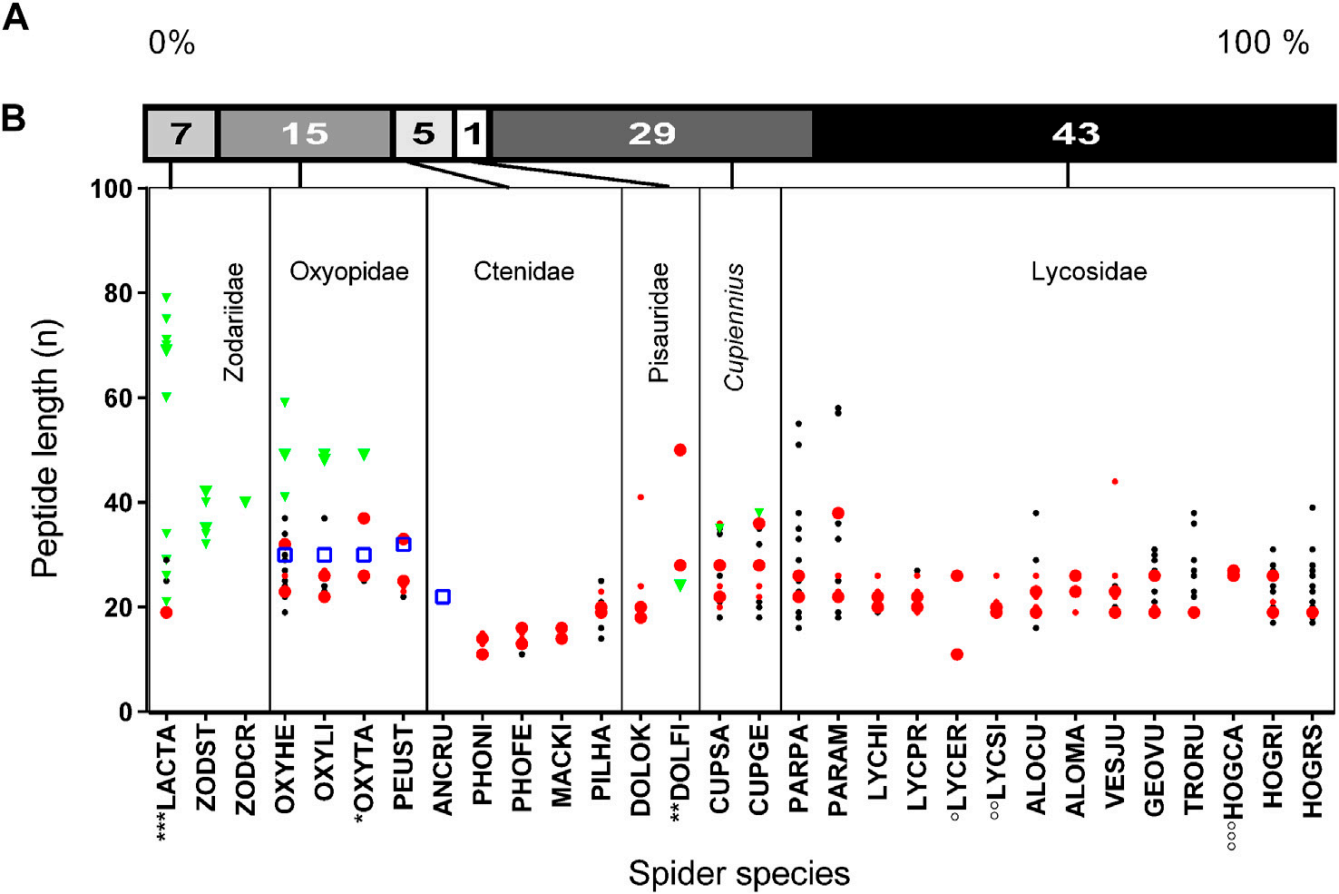
Length and abundance of peptides identified in simple and binary/complex precursors of different spider families. **(A)** Relative distribution of identified LPs in different spider families. **(B)** Spider families with the corresponding species where peptide lengths are given as amino acid residues per peptide (n). The abundance of peptides derived from simple precursors within a species is illustrated in big green triangles (>29%), and small green triangles (<29%). Peptides exhibiting a Rana box-like motif (Dubovskii et al., 2011) are in a blue squares. The abundance of peptides derived from binary/complex precursors within a species is illustrated in big red dots (>15%), small red dots (>10–14%), and black small dots (<10%). Data corresponding to *, **, ***, °, °°, °°° are from UniProtKB (Supplementary Table S3).

Through this study and with the recently published LPs from two *Cupiennius* species, our knowledge of such peptides and their cDNA structure in the venom of spiders increased from about 51 to about 812 records (Table 1), e.g., 831 records, taking also peptides into account, which are only identified on amino acid level so far. Besides *Cupiennius* species (29%), most LPs have been identified in lycosids (43%), and oxyopids (15%). The identified peptides can roughly be divided into short (< 30 amino acid residues), middle (30–60 amino acid residues) and long LPs (> 60 amino acid residues). Besides the known cytoinsectotoxins (Vassilevski et al., 2008) no further comparably long cationic peptides have been identified so far (Figure 5A,B).

Looking on the biochemical properties of LPs, most peptides are highly cationic due a large number of Lys and Arg within the sequences, arranged alternating with more hydrophobic amino acid residues. Strikingly, some peptides also contain N- and/or C-terminally well-defined hydrophobic parts, which are connected by a cationic middle part and, thus, result in amphipathic structures. The theoretical propensity of LPs, to build an α-helix in the presence of negatively charged membranes is given for many of them (Supplementary Table S4, S5). C-terminal amidation was predicted for many peptides among all investigated species. On the first run, no clear dependency between LPs amidation and linkers, or neighborhood to other peptides was apparent.

In oxyopids, many similar and two identical peptides were identified, whereas both *Cupiennius* species share several peptides. Strikingly, in lycosids several LP families are shared on amino acid level. The numbers of transcript families encoding different peptides in complex precursors are between one and four (Supplementary Table S2A–J). Except *Zodarion* species, pisaurids and *Ancylometes rufus*, in all other investigated species transcript families encoding short LPs were identified.

### Zodariidae

Posttranslational processing of LPs, identified in the venom and in the transcriptome of zodariid spiders, was for the first time described for latarcins and cyto-insectotoxins in *Lachesana tarabaevi.* Besides simple precursors, also binary and complex precursors were identified (Kozlov et al., 2006; Vassilevski et al., 2008). We investigated two *Zodarion* species that in contrast to the polyphagous *Lachesana*, are specialized ant hunter (Pekár et al., 2014; Pekár et al., 2018).

**FIGURE 6:**
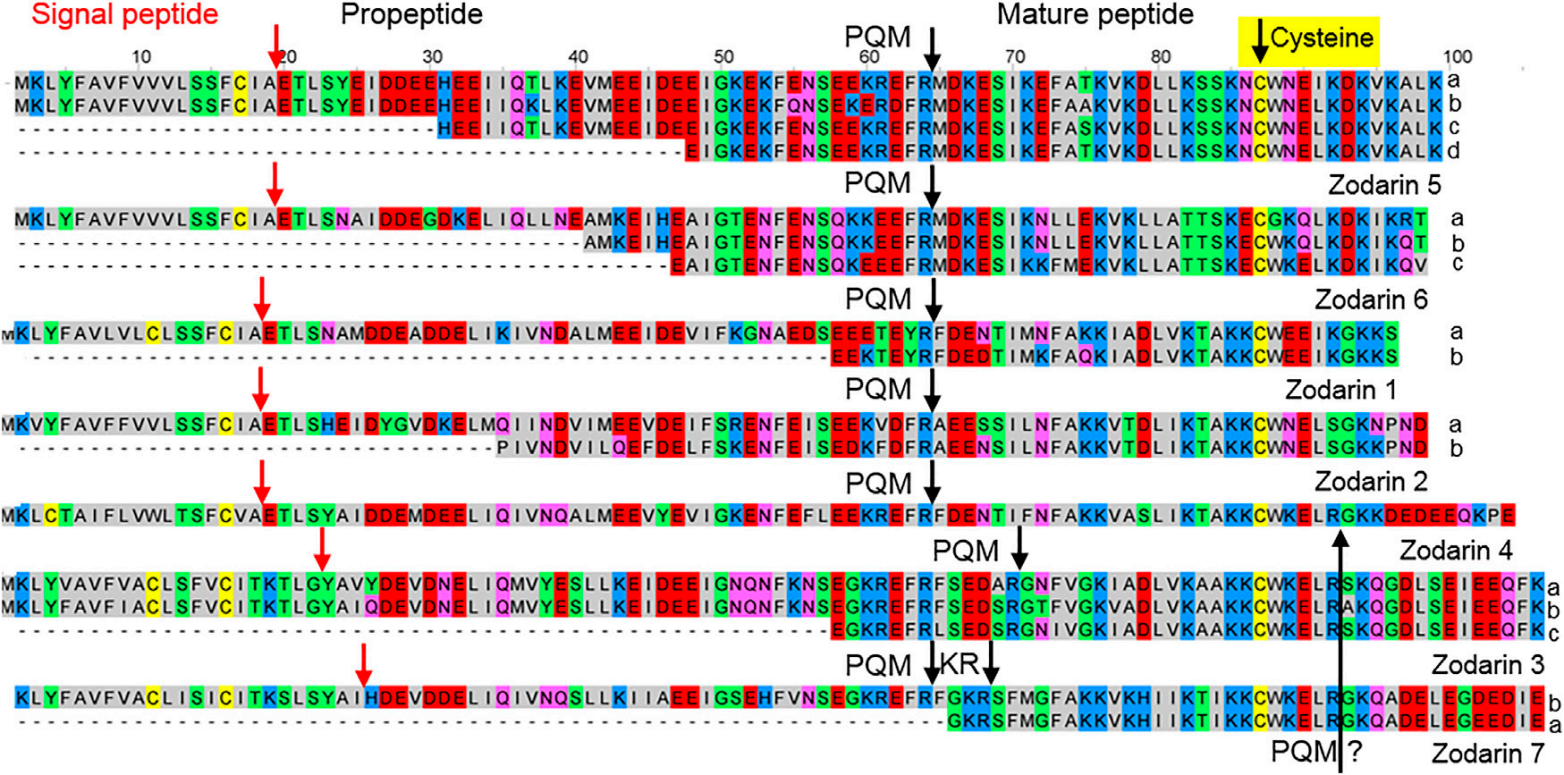
Overview of cysteine containing LPs identified in the venom gland transcriptome of *Zodarion cyrenaicum* (Zodarin 4) and *Zodarion styliferum* (Zodarins 1, 2, 3, 5, 6, 7). The cutting site (arrow) of the signal peptidase is colored in red and processing sites between propeptides and mature peptides are colored in black. Cationic amino acids are colored in blue, anionic amino acids in red, the corresponding C-terminal amid variants in pink, hydrophobic amino acids in black, Cys in yellow, and polar amino acids in green. A further possible PQM processing site was identified C-terminally in zodarins 3, 4, and 7 and is colored in black.

Searching the transcriptome with the BLAST function (E-value 0.0001) and using amino acid sequences of the above mentioned latarcins and cytoinsectotoxins as query, no related sequences were identified. Interestingly, reducing the E-value to 0.01 und using only the signal peptides together with the propeptides of the latarcin precursors, we identified simple precursor structures encoding cationic LPs. These peptides are characterized by a central Cys residue and 18–29% of their residues referring to positively charged amino acid residues, mainly Lys. They are composed of 32–42 amino acid residues with molecular masses of 3,715–4,953 Da and pIs between 7.9 and 9.7. After short signal peptides and longer propeptides, proteolytic cutting sites in form of a PQM motif are identified. However, additional KR-motifs as further cutting sites are recognized, which are located N-terminally or C-terminally of the PQM motif. Furthermore, in the C-terminal part of the mature zodarins 3, 4 and 7, a further PQM motif was identified, which allows the split-off of a more anionic sequence part, bringing linker-like structures to mind (Figure 6; Supplementary Table S2C, S4).

### Oxyopidae

Cytolytic peptides of oxyopids have been named oxyopinins 1, 2, 3, and 4 (Corzo et al., 2002; Dubovskii et al., 2011; Dubovskii et al., 2015). Transcripts of three species were investigated: *O. heterophthalmus*, *O. lineatus*, and *Peucetia striata*. Additionally, ENA deposited transcripts from *O. takobius* were included into our analysis. For naming and characterizing such peptides within transcripts, the recommended name oxyopinin will be used consequently for peptides from *Oxyopes* species and peucetin for *P. striata*.

**FIGURE 7:**
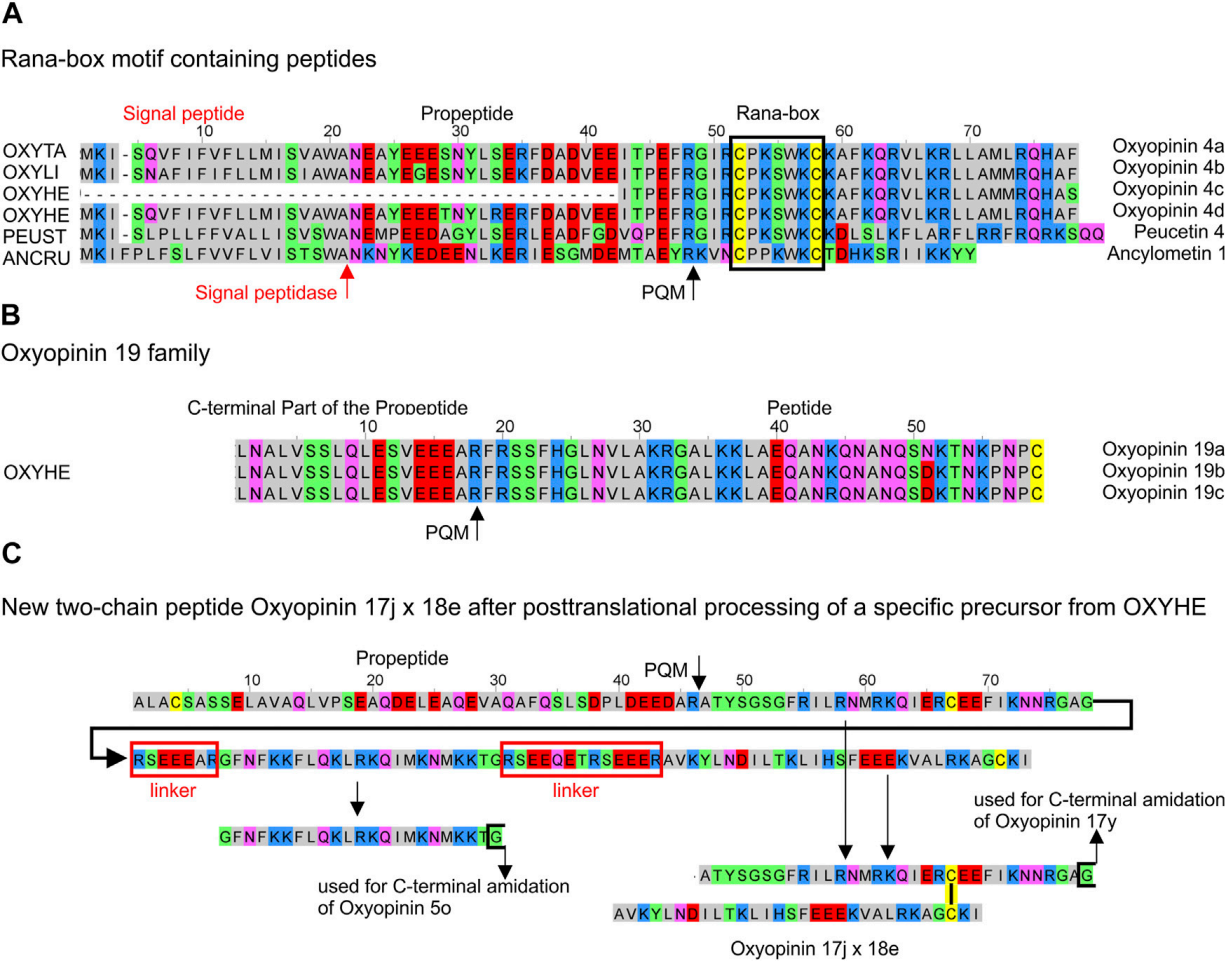
Overview of cysteine containing LPs identified in the venom gland transcriptomes of ctenids and oxyopids. **(A)** Rana-box like peptides identified in oxyopids such as *Oxyopes takobius* [F8J4S0, OXYTA], *O. lineatus* [OXYLI], *O. heterophthalmus* [OXYHE], *Peucetia striata* [PEUST], and in the ctenid *Ancylometes rufus* [ANCRU]. **(B)** Oxyopinin 19a, b, c with C-terminal Cys from *O. heterophthalmus* [OXYHE]. **(C)** Hypothetical processing of specific precursors identified in *O. heterophthalmus* [OXYHE] resulting in oxyopinin 5o and the two-chain peptide oxyopinin 17j x oxyopinin 18e. Cationic amino acids are colored in blue, anionic amino acids in red, the corresponding C-terminal amid variants in pink, hydrophobic amino acids in black, Cys in yellow, and polar amino acids in green.

Simples precursors, N-terminally composed of a signal peptide and a propeptide, encode diverse peptides of the oxyopinin 1 (5,069–5,290 Da), oxyopinin 4 (3,572–3,632 Da), oxyopinin 11 (6,525 Da), and oxyopinin 19 (4,524–4,553 Da) family. Two families attract special attention due to the presence of cysteines within the sequences. Oxyopinin 4 peptides are characterised by a Rana box-like motif, which shows after posttranslational modification an N-terminal disulfide bridge-stabilized loop (Dubovskii et al., 2011), also found for ancylometin 1 and peucetin 4. However, none of the Rana-box like peptides may play an important role in envenomation because they show low TPM values (200–1,376) and instead may belong to the innate immune system of spiders which also could explain the occurrence in a ctenid spider. Furthermore, oxyopinin 19a, b, c, exhibit cysteine as C-terminal amino acid residue (Figure 7A,B; Supplementary Table S2A, S4).

The available information on binary precursors in the transcriptomes of *Oxyopes* species is a bit contradictory. Whereas one transcript of oxyopinin 2 [A0A5J6SEB1] from *O. takobius* ends after the second peptide with a stop signal (TAA), pointing to a binary precursor structure, our transcript analysis of members of this family refer to two or three oxyopinin 2 peptides (4,091–4,161 Da), separated by different linkers within one transcript. Astonishingly, these linkers are composed of 24 amino acid residues, they are highly negatively charged (−7), and exhibit pIs about 3.9. The linkers amount to 2/3 of the length of oxyopinin 2 peptides, which are positively charged (+8) and a calculated pI is about 10.8. Furthermore, several complex precursor structures were identified, composed of mainly short peptides belonging to different oxyopinin families, always separated by short linkers (Supplementary Table S2A, S4).

From all investigated spider species, *Oxyopes heterophthalmus* with 19 different peptide families, shows the highest diversity of different LPs as well as of possible two-chain peptides (Figure 7C). Such peptides form posttranslationally a disulfide bridge and present similarities concerning structure and cDNA arrangement to the two-chain peptides (CsTx-16), identified in *Cupiennius salei* and *Cupiennius getazi* (Kuhn-Nentwig, 2021). In contrast to CsTx-16 peptides, only the first of both peptide chains (oxyopinin 17) in *O. heterophthalmus* is C-terminally amidated and is connected with a short linker to different variants of oxyopinin 5, continued by a longer linker, and followed afterwards by the second peptide chain (oxyopinin 18) and a stop signal (Figure 7C). Conspicuously, in oxyopids most propeptides and many linkers between different LPs are among the longest linker structures detected within all investigated spider species (Figure 4). Some of them exhibit a further PQM motif within the sequence.

The identified peptides of all three *Oxyopes* species are very similar in their amino acid sequences. However, in only one complex precursor of *O. heterophthalmus* and *O. lineatus*, two peptides, oxyopinin 8 (2,503 Da) and oxyopinin 12a (2,872 Da), are identical on amino acid level. Interestingly, oxyopinin 4 differs between *O. takobius* and *O. heterophthalmus* only in a C-terminally added Phe.

In contrast to *Oxyopes* species, transcripts of *Peucetia striata* encode mainly peptides with lengths between 22 and 33 amino acid residues except peucetin 2a, which comprises 57 amino acid residues. Peucetin 1 peptides (2,680–3,080 Da) are encoded in simple transcripts with short propeptides. The situation is similar with peucetin 4 (3,982 Da), which exhibits a Rana box-like motif (Figure 7A). Complex precursors can be divided in two major forms. Peucetin 2 (3,378–3,606 Da) peptides are connected to each other with linkers, which are 1.3 times longer than the peptides and some of them exhibit an additional PQM motif within their sequence. Peucetin 3 (3,105–3,167 Da), peucetin 5 (3,151–3,169 Da), and peucetin 6 (2,596 Da) are connected with short linkers composed of seven amino acid residues (Supplementary Table S2A, S4).

### Ctenidae

**FIGURE 8:**
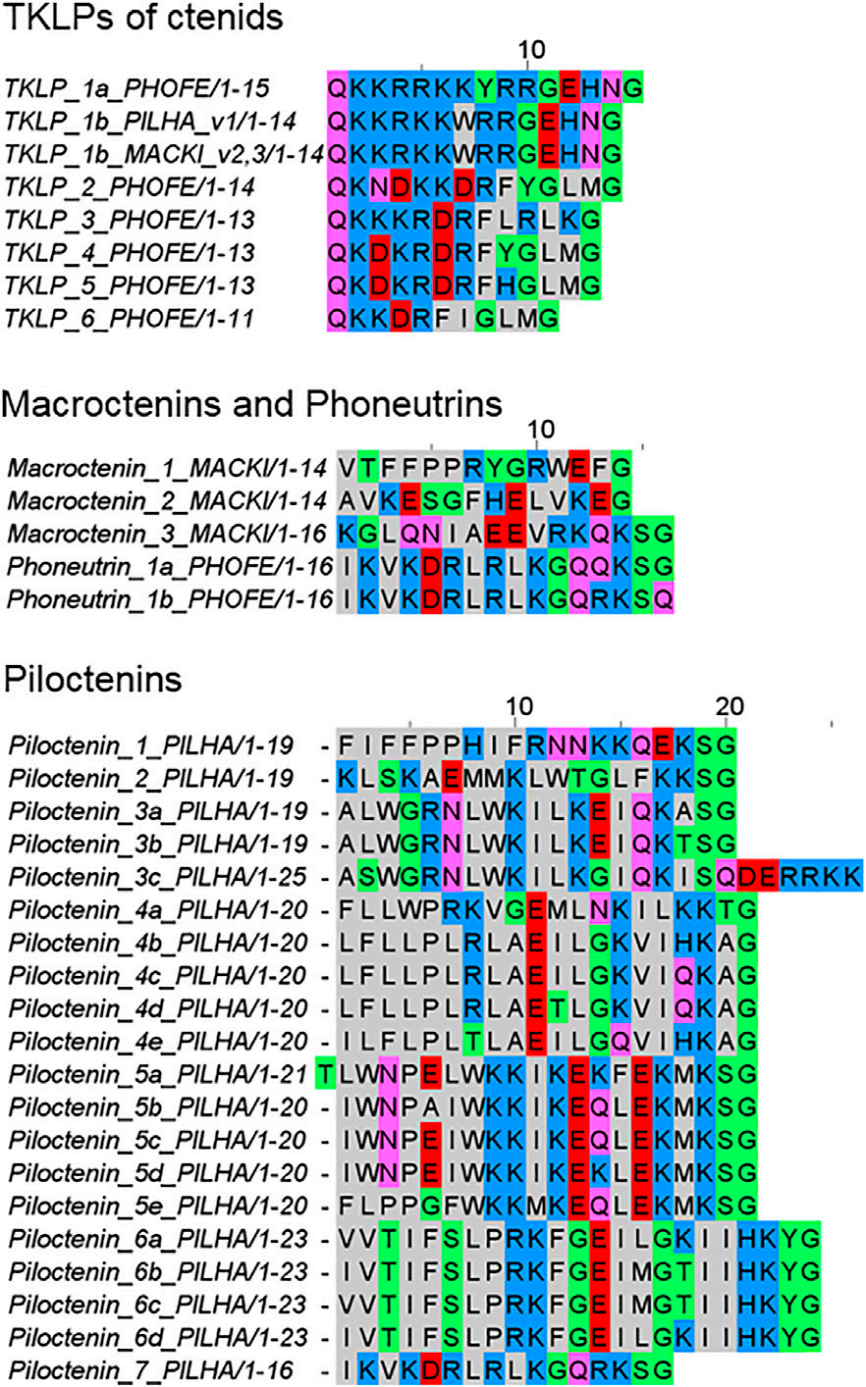
Overview of tachykinin-like peptides (TKLPs) and short LPs (macroctenins, phoneutrins, and piloctenins), identified in the venom gland transcriptomes of ctenids. PHOFE: *Phoneutria fera*, MACKI: *Macroctenus kingsleyi*, PILHA: *Piloctenus haematostoma*. Cationic amino acids are colored in blue, anionic amino acids in red, the corresponding C-terminal amid variants in pink, hydrophobic amino acids in black, and polar amino acids in green.

Surprisingly, three ctenids (*Phoneutria fera*, *Piloctenus haematostoma*, and *Macroctenus kingsleyi*) exhibit short LPs and tachykinin-like peptide (TKLP) sequences in their transcriptomes, which are encoded in complex precursor structures. All identified TKLPs are short peptides, composed of only 11–15 amino acid residues, with pIs of 9.5–11.7, and molecular masses of 1,293–1,941 Da. Further LPs, with so far unknown physiological functions, are only 16–25 amino acid residues long (pI 5.5–11.7, 1,530–3,039 Da) and are often characterized by a hydrophobic N-terminus and a more charged C-terminal part (Figure 8; Supplementary Table S2B, S4).

The composition of the complex precursor structures is comparable to those identified in *Cupiennius* (Kuhn-Nentwig, 2021) and *Lachesana tarabaevi* (Kozlov et al., 2006). TKLPs can be encodes in one complex precursor family (*P. fera*) or in two different complex precursor families together with other unknown short LPs (*P. haematostoma*, *M. kingsleyi*). In African ctenids only two TKLP 1b were identified, which are identical in their amino acid sequences, but show point mutations in their nucleotide sequences. TKLP 1b differs mainly from TKLP 1a of the South American ctenid *P. fera* by the deletion of one amino acid residue (Figure 8).

In *P. fera*, two different transcript families have been identified. One transcript family encodes phoneutrin 1a and 1b and at least five LPs are separated by short linkers. N-terminally of these peptides, every second amino acid residue is a positively charged residue (Lys and Arg) and more C-terminally, Gln is dominating. Only phoneutrin 1a is C-terminally amidated. Interestingly, the other transcript family encodes six different TKLPs, which are all C-terminally amidated. The peptides exhibit N-terminally a Gln residue, which is important for the formation of pyroglutamate, as described from purified TKLPs from the venom of *Phoneutria nigriventer* (Pimenta et al., 2005).

In contrast to *P. fera*, the two transcript families of *P. haematostoma* exhibit, after a very short propeptide, piloctenin 1 which is characterized by a hydrophobic N-terminus and two Pro in vicinity. The amidated C-terminus is positively charged. After a short linker, TKLP 1b is encoded and it is not clear, how the complex transcript is further built up. In further transcript families, LPs are separated by short linkers and belong to seven different piloctenin families (Figure 8; Supplementary Table S2B, S4).

*M. kingsleyi* exhibits two transcript families, in which TKLP 1b and only three different short LPs, macroctenins 1–3, are encoded together. TKLP 1b of both African ctenids are on amino acid residue level identical, but differ in several point mutations. Moreover, there are no obvious similarities between the identified LPs of ctenids and other spider families. However, piloctenin 7 shows a high amino acid sequence similarity with phoneutrin 1ab, but on nucleotide level more point mutations are present.

In the transcriptome of *Ancylometes rufus*, which also belongs to ctenids, we identified neither TKLPs nor LPs without cysteines. Astonishingly, a simple precursor was identified and encodes a peptide with two cysteines, comparable to oxyopinin 4, identified in the venom and transcriptome of Oxyopes takobius*.* It is tempting to speculate that this peptide, ancylometin 1 (22 aa, 2,736 Da, pI 9.8), forms posttranslationally a disulfide bridge-stabilized loop in N-terminal position and may act bactericidal as described for oxyopinin 4 (Figure 7A; Dubovskii et al., 2011).

### Pisauridae

**FIGURE 9:**
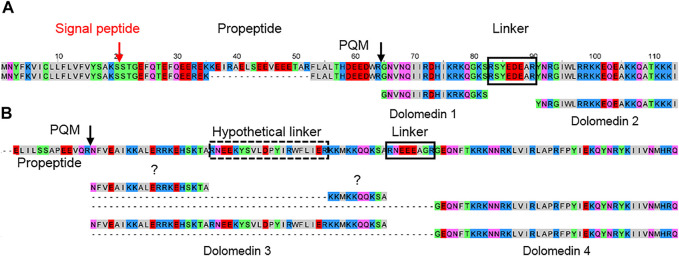
Hypothetical posttranslational processing of two peptide precursors of *Dolomedes okefinokensis*. **(A)** Peptide precursors of dolomedin 1 and 2. **(B)** Peptide precursor of dolomedin 3 and 4. After removing signal peptide and propeptide by specific proteases, the remaining peptide chain can be further processed by removing different peptide linkers (black boxes) through iPQM/PQM specific proteases resulting in dolomedin 1–4. Cationic amino acids are colored in blue, anionic amino acids in red, the corresponding C-terminal amid variants in pink, hydrophobic amino acids in black, and polar amino acids in green.

Pisaurids are a further family close to lycosids and, beside a few sequences in nucleotide databases, no information concerning LPs in their venom was available. We identified one possible binary precursor family in the transcriptome of *Dolomedes okefinokensis*, resulting in dolomedin 1 and 2. Both peptides are separated by a short linker (RSYEDEAR) and exhibit no C-terminal amidation. The precursors differ mainly in their propeptide region but show on amino acid sequence level identical signal peptides as well as LPs (Figure 9A).

In a second precursor family, the posttranslational processing of the obtained peptide chain by specific proteases into defined LPs and linkers is not so obviously. One processing site, where the linker RNEEEAGR corresponds to the linker length between dolomedin 1 and 2, is identified in the C-terminal part. N-terminally, a possible further linker could be RNEEKYSVLDPYIRWFLIER, but with 20 amino acid residues it is rather long and more hypothetical (Figure 9B; Supplementary Table S2D, S4).

Nevertheless, the obtained peptides dolomedin 3 (6,151.11 Da) and 4 (5,161.99 Da) are rather long with 41 and 50 amino acid residues, which is only known from latarcins and oxyopinins. In contrast to the here identified LPs in the *D. okefinokensis* transcriptome, we have not detected related LPs in the transcriptome of another pisaurid, *Pisaura mirabilis*.

### Lycosidae

Data about LPs in lycosids was restricted to three species and five peptides from *Lycosa singoriensis*, *L. erythrognatha*, and *Hogna carolinensis*. The peptides have been named lycotoxins (Yan and Adams, 1998), lycocitins (Budnik et al., 2004), or lycosins (Rádis-Baptista, 2021). We investigated eleven further lycosid species, identified a high number of LPs and classified them into six different peptide families. We named widespread LPs, shared with several lycosids genera, lycosin families 1–9. The other more genus or species specific peptide families were named after the genus where we identified most of those peptides, thus we named them alopecosins, geolycosins, hognins, pardosins, and trochosins (Supplementary Figure S4, Tables S5, S6).

From 352 identified LPs, 34 peptides are shared on amino acid sequence level with another lycosid species, four LPs are shared with three species and one and two peptides with four and five species. Between *Pardosa amentata* and *Pardosa palustris,* thus two species of the same genus, 26% of the LPs are identical. A similar case with 21% identical LPs was found between two further lycosids, *Trochosa ruricola* and *Alopecosa cuneata*. A special case concerned *Hogna radiata*: An Italian and a Spanish population comprised 16 identical LPs, thus differed for 11 and 17 LPs. In total, 256 species-specific and 96 shared peptides were identified for lycosids (Supplementary Table S2E–I, S5, S6).

**FIGURE 10:**
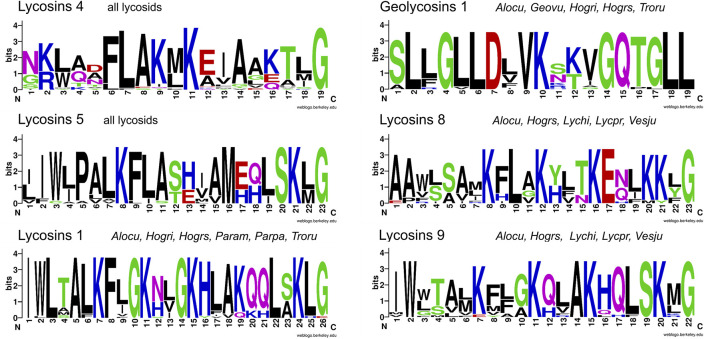
Overview of sequence logos of selected LP families of lycosids. Peptides of the lycosin 4 and 5 families are shared by all lycosids and peptides of the lycosin 1, geolycosin 1, lycosin 8, and lycosin 9 families are shared by only some species. The relative frequency of each amino acid residue at a certain position of different lycosid peptide families is given as a sequence logo. Cationic amino acids are colored in blue, anionic amino acids in red, the corresponding C-terminal amid variants in pink, hydrophobic amino acids in black, and polar amino acids in green. Alocu (*Alopecosa cuneata*), Geovu (*Geolycosa vultuosa*), Hogri/Hogrs (*Hogna radiata* Italy/Spain), Lychi (*Lycosa hispanica*), Lycpr (*Lycosa praegrandis*), Param (*Pardosa amentata*), Parpa (*Pardosa palustris*), Troru (*Trochosa ruricola*), and Vesju (*Vesubia jugorum*).

**FIGURE 11:**
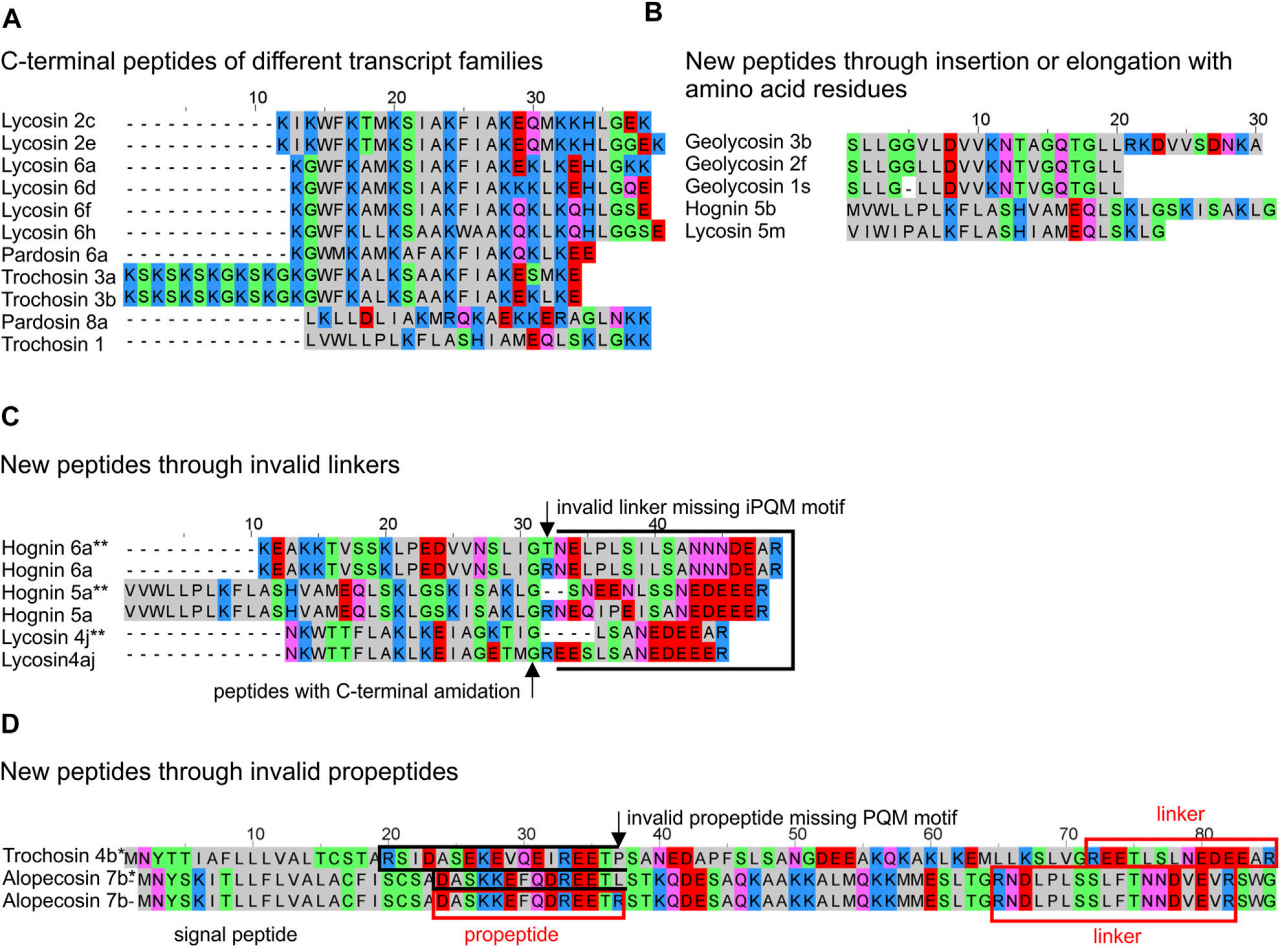
Overview of different features of LPs from lycosids resulting in new peptide structures. **(A)** C-terminal peptides of identified transcript families are not C-terminally amidated and differ in N-terminal mutations, within the peptides, and C-terminal mutations, but also by elongations and insertions. **(B)** New peptides occur through insertion within the peptide, and/or elongation of the C-terminal peptide part. **(C)** Invalid linkers C-terminally of LPs may results in fused peptides. **(D)** Invalid C-termini of propeptides may result in fused peptides. * theoretical N-terminally fused peptides, ** theoretical C-terminally fused peptides. Cationic amino acids are colored in blue, anionic amino acids in red, the corresponding C-terminal amid variants in pink, hydrophobic amino acids in black, and polar amino acids in green.

Two to four transcript families encode all LPs within one lycosid species and they are always constructed as complex precursor structures. Precursors can be assigned to two groups concerning their propeptide length. The most common propeptide lengths refer to 35–39 amino acid residues and encode, beside other peptide families, primarily the peptide families lycosins 4 and lycosins 5 in lycosids. Propeptides composed of less amino acid residues (22–27) mainly encode genus/species specific peptides. The high diversity of LPs within one peptide family is due to minor mutations at specific positions in the sequences, which may not affect the biological activity as shown in the sequence logos for the lycosin 1, 4, 5, 8, and 9 families (Figure 10; Supplementary Table S5). Moreover, N-terminal and C-terminal elongations as well as insertions and extensions of amino acid residues increase the number of peptide variants (Figure 11A,B).

Interestingly, mutations in the PQM region of propeptides, but also in the iPQM region of linkers result in new peptide structures. In *A. cuneata* and *T. ruricola*, a possible mutation concerning the C-terminal end of the propeptide results in a missing PQM cutting site. As consequence, this part of the propeptide may got fused with the first peptide leading to a shorter propeptide of only 10 or 14 amino acid residues and to peptides, which are characterized by a negatively charged N-terminus as shown for trochosin 4a-c* and alopecosin 7a*, b. Comparably, a mutation and/or deletion of the Arg residue of the iPQM motif of a linker, and C-terminal of a LP, results in an elongated peptide with a more polar or anionic C-terminus (Figure 11C,D).

Most LPs of lycosids exhibit molecular masses of 1,905–3,335 Da and have more or less cationic pIs (8.2–12), which corresponds mainly to 19 and 28 amino acid residues per peptide. However, shorter or longer peptides could also be identified (Figure 5B; Supplementary Table S2E–I, S5), mainly in the genus *Pardosa*. Here, pardosin families 10, 11, 12 are composed of 33–38 amino acid residues (3,477–4,320 Da) and the pardosin 13 members are composed of 55, 57, and 58 amino acid residues (5,721–6,307 Da). Pardosin 13 peptides are further characterized by two Cys, and one Cys terminates the peptides.

As mentioned for LPs of other spider families, most peptides are characterized by the repeated occurrence of Lys and/or Arg in every second, third or fourth amino acid position within the peptide. Such peptides are able to adopt an amphipathic structure in the presence of different membranes. The N-terminus of a LP can be hydrophobic or more polar and most peptides exhibit a C-terminal amidation. However, 22% of all LPs are not C-terminally amidated and most of them occur as C-terminal peptide of complex precursor structures (Figure 11A; Supplementary Table S2E–I, S5).

The ratio between hydrophobic and positively charged amino acids (percentage of hydrophobic amino acids divided by the percentage of positively charged amino acids) is between 4 and 9 for the peptide families geolycosins 1, trochosins 2, pardosins 4 and 5, hognin 5, and lycosin 5. The high content of hydrophobic amino acid residues is either located at the N-terminus (lycosin 5) or uniformly distributed over the entire peptide with a central positive charge as in geolycosins 1 (Figure 10).

Mainly in lycosids, we identified several processing mechanisms that result in new peptides: insertion/deletion of amino acid residues within a sequence, N- or C-terminal elongation of sequences (Figure 11A,B), but also invalid propeptides and linkers (Figure 11C,D).

**FIGURE 12:**
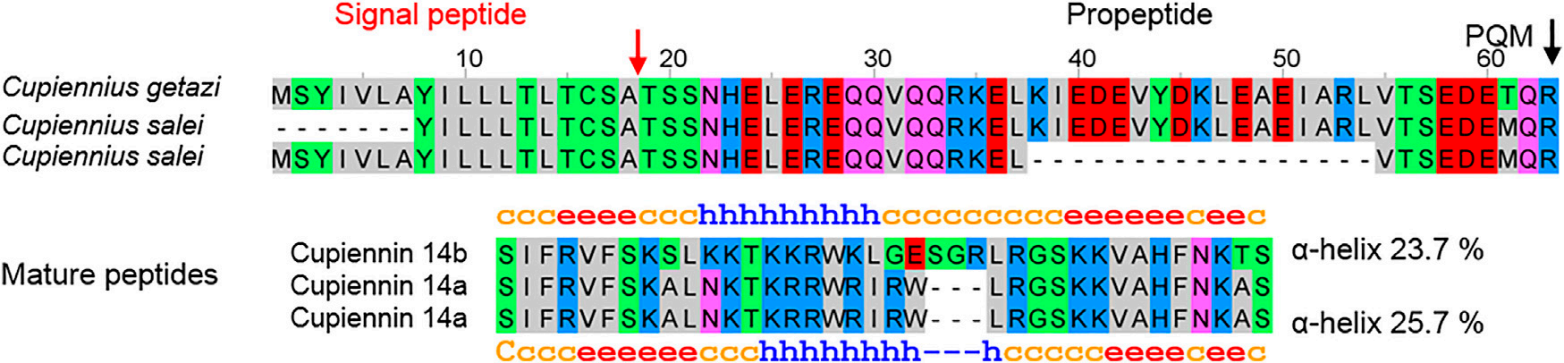
Simple precursors of LPs identified in the venom gland transcriptome of *Cupiennius salei* and *Cupiennius getazi* resulting after posttranslational processing in the mature peptides cupiennin 14a (*C. salei*) and cupiennin 14b (*C. getazi*).

No simple precursors were found in lycosid spiders, but in both *Cupiennius* species, we identified two related simple precursors, which encode after two different propeptides a highly cationic peptide of 35 amino acid residues (cupiennin 14a, 4,274.1 Da, pI 12.7), with 6 Arg and 6 Lys residues in the case of *C. salei*. Correspondingly, in *C. getazi*, the signal peptide and propeptide is on amino acid level very similar to the sequences of *C. salei*, but the highly cationic peptide is three amino acid residues longer (cupiennin 14b, 4,422.25 Da, pI 12.0) and contains 9 Lys and 4 Arg (Figure 12; Supplementary Table S2J). However, these precursors seem not to play a functionally important role taking the deep TPM values into account (CUPGE: 99, CUPSA: 72) and possibly may belong to the innate immune system of *Cupiennius* species.

## Discussion

Following the here presented state of knowledge, it is remarkable that LPs in spider venoms occur only in the RTA-clade, a rather modern branch of spiders. This allows the conclusion that LPs are a modern development among the main venom component groups and that the investment into LPs obviously boosted the toxicity of the venom and broadens the spectrum of possible prey. LPs destroy diverse membranes of cells or tissues and this probably allows to attack a wider spectrum of targets, compared to neurotoxins that address specific ion channels of muscle and nerve cells. Moreover, besides their own insecticidal activity, LPs enhance the toxicity of neurotoxins synergistically (Wullschleger et al., 2005; Dubovskii et al., 2015; Kuhn-Nentwig, 2021). Such a development towards more LPs in the venom, could indicate advantages in efficiency or economy, which is shown for many spider species in Table 2. Moreover, the correlation between the length of branch and the number of LPs in different spider species is highly significant (Figure 2). The content of LPs in the transcriptome of *C. salei* (454-sequencing technology) was earlier calculated to be about 25% (Kuhn-Nentwig et al., 2019; Kuhn-Nentwig, 2021) which is confirmed by 31% obtained by NGS. Strikingly, oxyopids (31–52%) show the highest content of LPs encoding contigs in the transcriptomes, followed by *Cupiennius* (31–44%), and with one exception, the lycosids (16–40%). Ctenids show low contents of such contigs in the transcriptomes (0.02–8%), except *Piloctenus haematostoma* (37%). For the here investigated pisaurid and zodariids LPs are probably functionally irrelevant.

**TABLE 2:** Overview of the percentage of LPs in the transcriptomes of different spiders.

Spider family	Spider species	Contigs related to LPs TPM (%) [A]^a^	All LPs TPM (%) [B]^b^	[B]/[A]
				
Lycosidae	*Alopecosa cuneata*	27.4	76.4	2.8
	*Alopecosa marikovskyi*	1.1	1.8	1.6
	*Geolycosa vultuosa*	38.5	74.1	1.9
	*Hogna radiata* (Spain)	19.9	52.0	2.6
	*Hogna radiata* (Italy)	23.5	45.5	1.9
	*Lycosa hispanica*	40.3	112.6	2.8
	*Lycosa praegrandis*	39.9	97.2	2.4
	*Pardosa amentata*	19.4	35.8	1.8
	*Pardosa palustris*	16.1	25.3	1.6
	*Trochosa ruricola*	27.5	43.3	1.6
	*Vesubia jugorum*	30.8	78.2	2.5
Trechaleidae	*Cupiennius getazi*	44.2	71.0	1.6
	*Cupiennius salei*	31.3	38.9	1.2
Ctenidae	*Ancylometes rufus*	0.021	0.020	0.95
	*Macroctenus kingsleyi*	0.3	0.6	1.8
	*Phoneutria fera*	7.9	20	2.5
	*Piloctenus haematostoma*	36.6	78.1	2.1
Oxyopidae	*Oxyopes heterophthalmus*	52.1	52.6	1.0
	*Oxyopes lineatus*	31.2	24.5	0.8
	*Peucetia striata*	47.8	54.4	1.1
Pisauridae	*Dolomedes okefinokensis*	1.2	0.7	0.6
Zodariidae	*Zodarion styliferum*	2.8	1.4	0.5
	*Zodarion cyrenaicum*	0.2	0.2	1
				

^a^ TPM values are calculated for all individual contigs encoding different LPs structures and expressed as percentage of TPM corresponding to each transcriptome.

^b^ The amount (TPM %) of all identified LPs in a transcriptome is given as sum of the corresponding TPM values of the corresponding contigs, which allows a conclusion about the relative abundance of each LP. Only complete peptides, with C-terminal amidation if present, were used for the calculation.

To evaluate the impact of complex precursors in different transcriptomes, we have generate the quotient ([B]/[A]) between all counted LPs [B] (TPM %) and all LPs containing contigs in a transcriptome [A] (TPM %). The ratio should be about one, if one contig encodes one LP (Table 2). The ratio B/A between the TPM (%) values of identified LPs in a transcriptome and the TPM (%) belonging to the corresponding contigs, shows roughly the minimum impact of complex precursors in lycosids (1.6–2.8 fold), *Cupiennius* (1.2–1.6 fold), and ctenids (1–2.5 fold). Here, one precursor structure encodes several distinct or identical LPs. This is different in oxyopids (0.8–1.1 fold), where the balance is more in favor of simple/binary precursor structures and the most present LPs are those belonging to the oxyopinin 2 family (OXYLI: 144,690 TPM; OXYHE: 309,038 TPM) (Supplementary Table S7).

**FIGURE 13:**
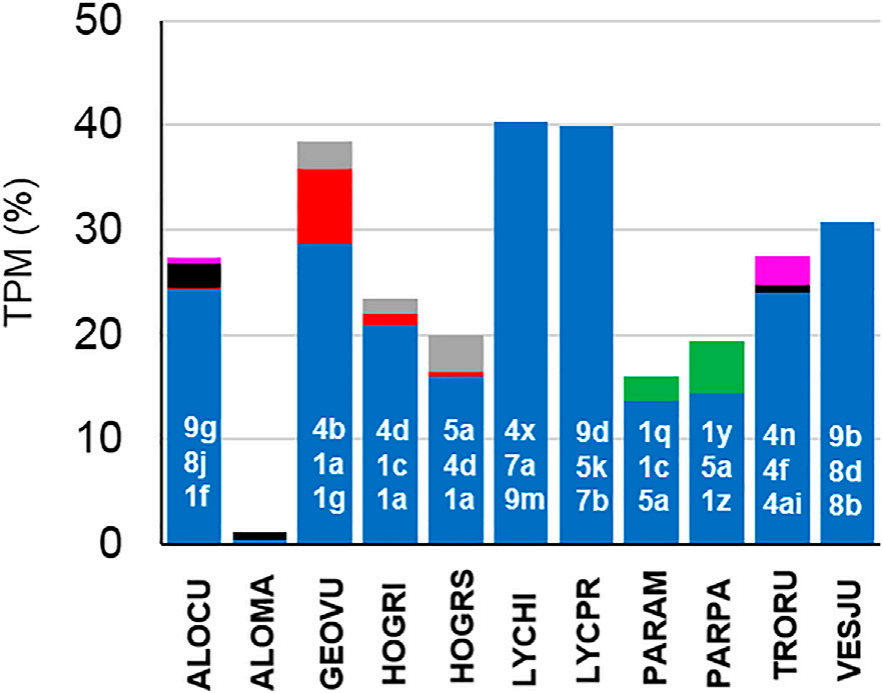
Comparison of the frequency of occurrence of LP families in lycosids species. Lycosin families are colored in blue, pardosins in green, trochosins in pink, hognins in gray, and geolycosins in red. Individual members of the three most abundant lycosin families [highest Transcripts Per Kilobase Million (TPM) values] are given in white numbers (Supplementary Table S7).

Focusing on lycosids, the most significant and probably originally peptide family identified in all lycosid transcriptomes is the lycosin (1–9) family (Figure 13) with 144 individual peptides out of 183 identified lycosin 1–9 structures. Members of this peptide family occur in all lycosids, suggesting its importance, while genus specific peptide families as pardosins, trochosins, geolycosins, and hognins may play an underpart in the envenomation process. In lycosids, one of the youngest spider families, LPs are most widespread and somehow similar in all investigated species. Contrary, in oxyopids their LP families seem to be more genus specific, because LPs identified in *Peucetia striata*, are not very similar to LPs from *Oxyopes* species, with the exception of the Rana-box like peptides, which were also detected in a ctenid spider. The low appearance in the transcriptomes may point to another function of these peptides as part of the innate immunity, which may be only upregulated after a microbial invasion and therefore only available in traces in venom glands.

If LPs as a major venom component had been invented at the basis of the RTA-clade, one would expect that all included families should possess them. However, we found LPs in only five out of 17 investigated spider families of the RTA-clade. It remains enigmatic, why we could not detect LPs in Thomisidae, sister family to Oxyopidae where they occur in high numbers. Also in Trechaleidae (*Trechaleoides biocellata*), for a long time considered to belong to Pisauridae, now a sister family to Lycosidae, we did not reveal any LPs, whereas they occur in high diversity in *Cupiennius*, in all Lycosidae and at very low level in Pisauridae. Therefore, we are not convinced that *Cupiennius* is correctly placed in Trechaleidae.

In Zodariidae only *Lachesana tarabaevi* possess a high portion of different LPs with confirmed cytolytical activities (Dubovskii et al., 2015) whereas two *Zodarion* species exhibit only a few peptides which are encoded in LP precursors with unknown activities. They do not seem to be functionally very important when taking the TPM values into account (ZODST all zodarins: 13,661 TPM, ZODCY zodarin 4: 1,945 TPM) (Supplementary Table S7).

We found mainly weak similarities between the LP structures of different spider families and they show a remarkable own development of LPs. However, some general pattern can be found as they are mainly encoded in complex precursor structures, sometimes also in simple precursor structures (oxyopids). Furthermore, obvious features are the repeated occurrence of cationic amino acids in every third or fourth position of different long peptides, hydrophobic N-terminal or C-terminal parts, and the propensity to form α-helices. Additionally, there are short peptides with a more central cationic part and more hydrophobic N- or C-termini, or a well-defined hydrophobic part within a cationic charged peptide, showing low propensity to form α-helices. The occurrence of insecticidally acting cationic two-chain peptides as identified in *Cupiennius* species (Kuhn-Nentwig, 2021) and proposed for *O. heterophthalmus* point to parallel developments within RTA-clade spider families using complex precursors structures. Until now, on amino acid level, identical LPs are only found within lycosids, the genus *Cupiennius* and rarely within ctenids and oxyopids.

This could indicate that the overall “idea” of LPs as venom component became available with zodariids at the basis of the RTA-clade, but the realization happened only in a few families. Alternatively, one could postulate the invention of LPs at the basis of the RTA-clade and a subsequent series of losses of this invention. Then, however, it would be enigmatic why such families should have lost such a successful innovation.

This thought leads to a more general point. It is possible that, by transcriptome analysis, components cannot be found because they are due to unknown circumstances not expressed. It is also possible that they can only be found at very low expression levels or that they occur in a modified or truncated version, thus they are overlooked. Tachykinin-like peptides (TKLPs) may indicate this in an impressive manner. They were first detected in the venom of *Phoneutria nigriventer* (Ctenidae) by classical methods (Pimenta et al., 2005), but could not be confirmed in several follow-up transcriptome studies (Diniz et al., 2018; Paiva et al., 2019). In the here presented analysis, we identified different TKLPs beside two low expressed LPs in 7.9% of all contigs in the transcriptome of *Phoneutria fera*, which are partially identical to the above described peptides of *P. nigriventer*. In the African ctenid *Piloctenus haematostoma*, the expression of TKLPs is reduced in favor of complete new peptide structures (piloctenin families 1–7) counting to 37% of all contigs in the transcriptome. Given such problems, we assume that TKLPs could be widespread in ctenid spider venoms, but were not detected so far. The same conclusion can also be drawn for LPs in general. Here, for the first time we show that, besides membrane active LPs, also other bioactive peptides like TKLPs underlie the same production mode as LPs in spider venom glands.

This tachykinin example shows that transcriptome data analysis may or may not yield a given result. Therefore, in a next step, it would be meaningful to validate the here obtained next generation sequencing data, especially data concerning complex peptide precursors, by third generation sequencing techniques, such as Pacific Bioscience (PacBio), and/or Oxford Nanopore Technologies (Nanopore). These techniques provide much longer read length and enable full-length mRNA sequencing (Giordano et al., 2017; Bayega et al., 2018). Together with top down proteomics of single spider venoms by online-HPLC coupled with Fourier-transform ion cyclotron resonance or Fourier-transform orbitrap mass spectrometry analysis could confirm single LPs (Melani et al., 2017; Ghezellou et al., 2018). Furthermore, genomic sequencing of such complex precursor structures could shed some light on the mechanism behind the high diversity of LPs. So far, in depth-investigations of the insecticidal and cytolytic activities of such peptides have mainly be performed for cupiennins (Kuhn-Nentwig, 2021) and latarcins (Dubovskii et al., 2015), a few data are also available for lycosins (Yan and Adams, 1998; Melo-Braga et al., 2020) and oxyopinins (Corzo et al., 2002). A next step should be the synthesis of the core peptides here presented and a detailed analysis of possible effects on different membrane systems, cell types, as well as on insects.

The evolutionary history of LPs in spider venoms is still unknown. Despite intensive analysis of different tissue specific transcriptomes (muscles, hemocytes, and nerves) of *Cupiennius salei,* searching for peptides and their precursors that might have been convergently recruited into the venom, as shown for a hyperglycemic hormone for other arthropods, failed in spiders (Undheim et al., 2015). For all these reasons mentioned above, we recommend, to supplement transcriptome studies with genome analyses.

The tremendous diversity of LPs is mainly encoded in complex precursor structures. The mechanisms behind this are gene duplication, diversification and intragene duplication as mentioned already for neurotoxins (Pineda et al., 2020). Such mechanisms may explain the occurrence of new peptide variants in different transcriptomes of the same species, as shown for *Hogna radiata* and *Cupiennius salei* (Kuhn-Nentwig, 2021). Specific for spider DNA is the occurrence of long introns and short exons (Sanggaard et al., 2014), which may results in alternative splicing of such genes. Further mechanisms as the induction of a hypervariability-generating mechanism and gene-based combinatorial peptide library strategies (Sollod et al., 2005) could be additional driving forces behind this diversity.

In summary, some modern spider use complex precursor structures for the fast and economic production of a tremendous variety of different membrane active LPs (Kuhn-Nentwig et al., 2011b; Dubovskii et al., 2015), but also for TKLPs and other new peptides, where the targets still have to be identified in the future. The here presented specific expression strategy and the knowledge of possible PQM proteases (Langenegger et al., 2018; Langenegger et al., 2019) important for the processing of such precursors, indicates new application strategies and is, therefore, of great interest for the pharmaceutical industry (Robinson et al., 2017; Reis et al., 2018; Saez and Herzig, 2019; Melo-Braga et al., 2020).

## Data Availability

The data for this study have been deposited in the European Nucleotide Archive (ENA) at EMBL EBI under accession number PRJEB44724 (https://www.ebi.ac.uk/ena/browser/view/PRJEB44724). The original contributions presented in the study are included in Supplementary Material.
