# Supplementary material for: Linear Peptides—A Combinatorial Innovation in the Venom of Some Modern Spiders
Source: Front Mol Biosci. 2021 Jul 6;8:705141. doi: 10.3389/fmolb.2021.705141 (PMC8290080; doi:10.3389/fmolb.2021.705141)
Supplement: Supplementary file 1 [file DataSheet1.zip › Supplementary Figure S1.PDF]

Linear Peptides – a Combinatorial Innovation in the Venom of Some Modern Spiders

Frontiers in Molecular Biosciences section **Cellular Biochemistry**

Lucia Kuhn-Nentwig et al.
Institute of Ecology and Evolution, University of Bern, Baltzerstrasse 6, 3012 Bern, Switzerland
lucia.kuhn@iee.unibe.ch

Supplementary Figure S1. Evolutionary analysis of signal peptides of simple, binary and complex precursor structures encoding LPs (Maximum Likelihood method and JTT matrix-based model).

Included are signal peptides of simple precursors encoding neurotoxins with an ICK motif as well as neurotoxins featuring a highly cationic α-helical motif (N-terminal) and an ICK motif (C-terminal: spiderines*).

*Vassilevski, A.A., Sachkova, M.Y., Ignatova, A.A., Kozlov, S.A., Feofanov, A.V., and Grishin, E.V. (2013). Spider toxins comprising disulfide-rich and linear amphipathic domains: a new class of molecules identified in the lynx spider *Oxyopes takobius*. *FEBS J* 280**,** 6247-6261.

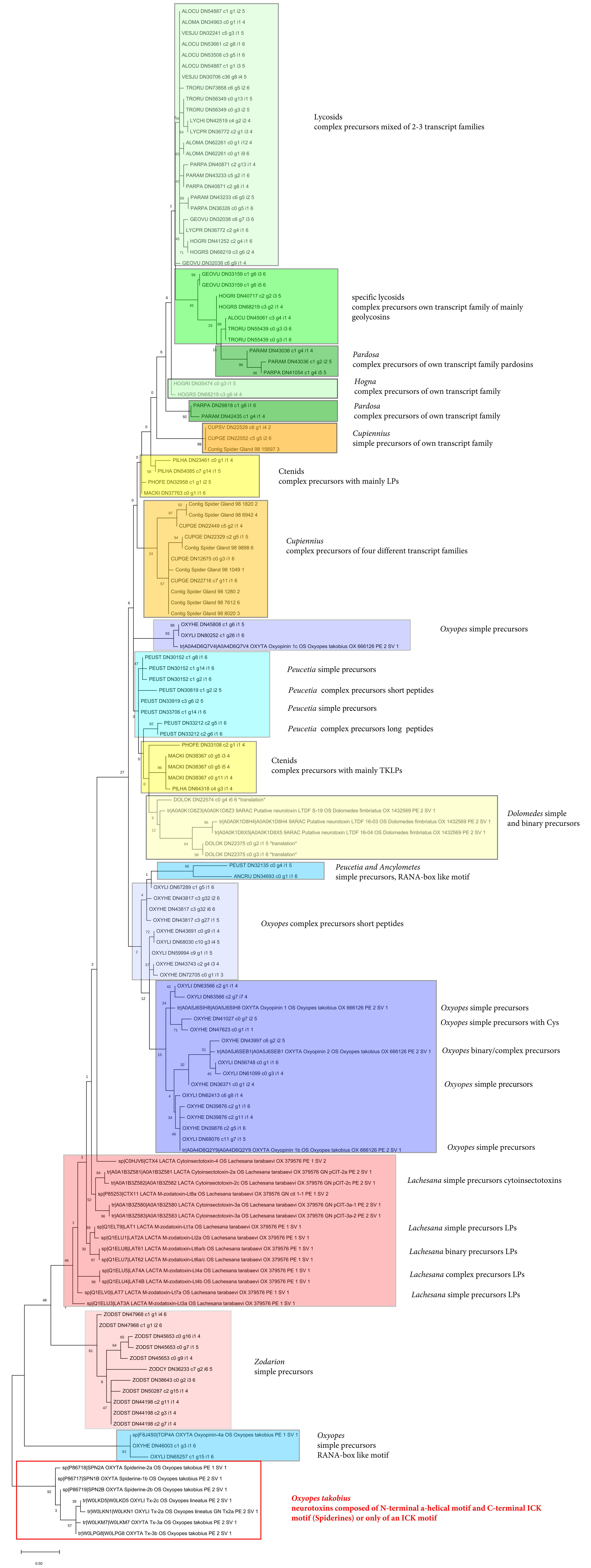
ALOCU DN54887 c1 g1 i2 5
ALOMA DN34963 c0 g1 i1 4
VESJU DN32241 c5 g3 i1 5
ALOCU DN53661 c2 g8 i1 6
ALOCU DN53508 c3 g5 i1 6
ALOCU DN54887 c1 g1 i3 5
VESJU DN30706 c36 g8 i4 5
TRORU DN73858 c6 g5 i2 6
TRORU DN56349 c0 g13 i1 5
TRORU DN56349 c0 g3 i2 5
LYCHI DN42519 c4 g2 i2 4
LYCPR DN36772 c2 g1 i3 4
ALOMA DN62261 c0 g1 i12 4
ALOMA DN62261 c0 g1 i9 6
PARPA DN40871 c2 g13 i1 4
PARAM DN43233 c5 g2 i1 6
PARPA DN40871 c2 g8 i1 4
PARAM DN43233 c6 g5 i2 5
PARPA DN36326 c0 g5 i1 6
GEOVU DN32038 c6 g7 i3 6
LYCPR DN36772 c2 g4 i1 6
HOGRI DN41252 c2 g4 i1 6
HOGRS DN68219 c3 g6 i2 4
GEOVU DN32038 c6 g9 i1 4
Lycosids
complex precursors mixed of 2-3 transcript families
GEOVU DN33159 c1 g6 i3 6
GEOVU DN33159 c1 g6 i5 6
HOGRI DN40717 c2 g2 i3 5
HOGRS DN68219 c3 g2 i1 4
ALOCU DN45061 c3 g4 i1 4
TRORU DN55439 c0 g3 i3 6
TRORU DN55439 c0 g3 i1 6
specific lycosids
complex precursors own transcript family of mainly geolycosins
PARAM DN43036 c1 g4 i1 4
PARAM DN43036 c1 g2 i2 5
PARPA DN41054 c1 g4 i5 5
Pardosa
complex precursors of own transcript family pardosins
HOGRI DN35474 c0 g3 i1 5
HOGRS DN68219 c3 g6 i4 4
Hogna
complex precursors of own transcript family
PARPA DN29818 c1 g8 i1 6
PARAM DN42435 c1 g4 i1 4
Pardosa
complex precursors of own transcript family
CUPSV DN22529 c6 g1 i4 2
CUPGE DN22552 c5 g5 i2 6
Contig Spider Gland 98 15697 3
Cupiennius
simple precursors of own transcript family
PILHA DN23461 c0 g1 i1 4
PILHA DN54085 c7 g14 i1 5
PHOFE DN32958 c1 g1 i2 5
MACKI DN37763 c0 g1 i1 6
Ctenids
complex precursors with mainly LPs
Contig Spider Gland 98 1820 2
Contig Spider Gland 98 6942 4
CUPGE DN22449 c5 g2 i1 4
CUPGE DN22329 c2 g5 i1 5
Contig Spider Gland 98 9898 6
CUPGE DN12675 c0 g3 i1 6
Contig Spider Gland 98 1049 1
CUPGE DN22716 c7 g11 i1 6
Contig Spider Gland 98 1280 2
Contig Spider Gland 98 7612 6
Contig Spider Gland 98 8020 3
Cupiennius
complex precursors of four different transcript families
OXYHE DN45808 c1 g6 i1 5
OXYLI DN80252 c1 g26 i1 6
tr|A0A4D6Q7V4|A0A4D6Q7V4 OXYTA Oxyopinin 1c OS Oxyopes takobius OX 666126 PE 2 SV 1
Oxyopes simple precursors
PEUST DN30152 c1 g8 i1 6
PEUST DN30152 c1 g14 i1 6
PEUST DN30152 c1 g2 i1 6
PEUST DN30819 c1 g2 i2 5
PEUST DN33919 c3 g6 i2 5
PEUST DN33706 c1 g14 i1 6
PEUST DN33212 c2 g5 i1 6
PEUST DN33212 c2 g6 i1 6
Peucetia simple precursors
Peucetia complex precursors short peptides
Peucetia simple precursors
Peucetia complex precursors long peptides
PHOFE DN33108 c2 g1 i1 4
MACKI DN38367 c0 g5 i3 4
MACKI DN38367 c0 g5 i5 4
MACKI DN38367 c0 g11 i1 4
PILHA DN64318 c4 g3 i1 4
Ctenids
complex precursors with mainly TKLPs
DOLOK DN22574 c0 g4 i6 6 *translation*
tr|A0A0K1D8Z3|A0A0K1D8Z3 9ARAC Putative neurotoxin LTDF S-19 OS Dolomedes fimbriatus OX 1432569 PE 2 SV 1
tr|A0A0K1D8H4|A0A0K1D8H4 9ARAC Putative neurotoxin LTDF 16-03 OS Dolomedes fimbriatus OX 1432569 PE 2 SV 1
tr|A0A0K1D8X5|A0A0K1D8X5 9ARAC Putative neurotoxin LTDF 16-04 OS Dolomedes fimbriatus OX 1432569 PE 2 SV 1
DOLOK DN22375 c0 g2 i1 5 *translation*
DOLOK DN22375 c0 g3 i1 6 *translation*
Dolomedes simple and binary precursors
PEUST DN32135 c0 g4 i1 5
ANCRU DN34693 c0 g1 i1 6
Peucetia and Ancylometes
simple precursors, RANA-box like motif
OXYLI DN67289 c1 g5 i1 6
OXYHE DN43817 c3 g32 i2 6
OXYHE DN43817 c3 g32 i6 6
OXYHE DN43817 c3 g27 i1 5
OXYHE DN43691 c0 g9 i1 4
OXYLI DN68030 c10 g3 i4 5
OXYLI DN59994 c9 g1 i1 5
OXYHE DN43743 c2 g4 i3 4
OXYHE DN72705 c0 g1 i1 3
Oxyopes complex precursors short peptides
OXYLI DN63566 c2 g1 i1 4
OXYLI DN63566 c2 g7 i7 4
tr|A0A5J6SIH8|A0A5J6SIH8 OXYTA Oxyopinin 1 OS Oxyopes takobius OX 666126 PE 2 SV 1
OXYHE DN41027 c0 g7 i2 5
OXYHE DN47623 c0 g1 i1 1
OXYHE DN43997 c6 g2 i2 5
tr|A0A5J6SEB1|A0A5J6SEB1 OXYTA Oxyopinin 2 OS Oxyopes takobius OX 666126 PE 2 SV 1
OXYLI DN56748 c0 g1 i1 6
OXYLI DN61099 c0 g3 i1 4
OXYHE DN36371 c0 g1 i2 4
OXYLI DN62413 c6 g8 i1 4
OXYHE DN39876 c2 g1 i1 6
OXYHE DN39876 c2 g11 i1 4
OXYHE DN39876 c2 g5 i1 6
OXYLI DN68076 c11 g7 i1 5
tr|A0A4D6Q2Y9|A0A4D6Q2Y9 OXYTA Oxyopinin 1b OS Oxyopes takobius OX 666126 PE 2 SV 1
Oxyopes simple precursors
Oxyopes simple precursors with Cys
Oxyopes binary/complex precursors
Oxyopes simple precursors
Oxyopes simple precursors
sp|C0HJV6|CTX4 LACTA Cytoinsectotoxin-4 OS Lachesana tarabaevi OX 379576 PE 1 SV 2
tr|A0A1B3Z581|A0A1B3Z581 LACTA Cytoinsectotoxin-2a OS Lachesana tarabaevi OX 379576 GN pCIT-2a PE 2 SV 1
tr|A0A1B3Z582|A0A1B3Z582 LACTA Cytoinsectotoxin-2c OS Lachesana tarabaevi OX 379576 GN pCIT-2c PE 2 SV 1
sp|P85253|CTX11 LACTA M-zodatoxin-Lt8a OS Lachesana tarabaevi OX 379576 GN cit 1-1 PE 1 SV 2
tr|A0A1B3Z580|A0A1B3Z580 LACTA Cytoinsectotoxin-3a OS Lachesana tarabaevi OX 379576 GN pCIT-3a-1 PE 2 SV 1
tr|A0A1B3Z583|A0A1B3Z583 LACTA Cytoinsectotoxin-3a OS Lachesana tarabaevi OX 379576 GN pCIT-3a-2 PE 2 SV 1
sp|Q1ELT9|LAT1 LACTA M-zodatoxin-Lt1a OS Lachesana tarabaevi OX 379576 PE 1 SV 1
sp|Q1ELU1|LAT2A LACTA M-zodatoxin-Lt2a OS Lachesana tarabaevi OX 379576 PE 1 SV 1
sp|Q1ELU8|LAT61 LACTA M-zodatoxin-Lt6a/b OS Lachesana tarabaevi OX 379576 PE 1 SV 1
sp|Q1ELU7|LAT62 LACTA M-zodatoxin-Lt6a/c OS Lachesana tarabaevi OX 379576 PE 1 SV 1
sp|Q1ELU5|LAT4A LACTA M-zodatoxin-Lt4a OS Lachesana tarabaevi OX 379576 PE 1 SV 1
sp|Q1ELU4|LAT4B LACTA M-zodatoxin-Lt4b OS Lachesana tarabaevi OX 379576 PE 1 SV 1
sp|Q1ELV0|LAT7 LACTA M-zodatoxin-Lt7a OS Lachesana tarabaevi OX 379576 PE 1 SV 1
sp|Q1ELU3|LAT3A LACTA M-zodatoxin-Lt3a OS Lachesana tarabaevi OX 379576 PE 1 SV 1
Lachesana simple precursors cytoinsectotoxins
Lachesana simple precursors LPs
Lachesana binary precursors LPs
Lachesana complex precursors LPs
Lachesana simple precursors LPs
ZODST DN47968 c1 g1 i4 6
ZODST DN47968 c1 g1 i2 6
ZODST DN45653 c0 g16 i1 4
ZODST DN45653 c0 g7 i1 5
ZODST DN45653 c0 g9 i1 4
ZODCY DN36233 c7 g2 i6 5
ZODST DN38643 c0 g2 i3 6
ZODST DN50287 c2 g15 i1 4
ZODST DN44198 c2 g11 i1 4
ZODST DN44198 c2 g3 i1 4
ZODST DN44198 c2 g7 i1 4
Zodarion
simple precursors
sp|F8J4S0|TOP4A OXYTA Oxyopinin-4a OS Oxyopes takobius PE 1 SV 1
OXYHE DN46003 c1 g3 i1 6
OXYLI DN65257 c1 g15 i1 6
Oxyopes
simple precursors
RANA-box like motif
sp|P86718|SPN2A OXYTA Spiderine-2a OS Oxyopes takobius PE 1 SV 1
sp|P86717|SPN1B OXYTA Spiderine-1b OS Oxyopes takobius PE 2 SV 1
sp|P86719|SPN2B OXYTA Spiderine-2b OS Oxyopes takobius PE 2 SV 1
tr|W0LKD5|W0LKD5 OXYLI Tx-2c OS Oxyopes lineatus PE 2 SV 1
tr|W0LKN1|W0LKN1 OXYLI Tx-2a OS Oxyopes lineatus GN Tx2a PE 2 SV 1
tr|W0LKM7|W0LKM7 OXYTA Tx-3a OS Oxyopes takobius PE 2 SV 1
tr|W0LPG8|W0LPG8 OXYTA Tx-3b OS Oxyopes takobius PE 2 SV 1
Oxyopes takobius
neurotoxins composed of N-terminal a-helical motif and C-terminal ICK motif (Spiderines) or only of an ICK motif
0.50
