# Supplementary material for: Linear Peptides—A Combinatorial Innovation in the Venom of Some Modern Spiders
Source: Front Mol Biosci. 2021 Jul 6;8:705141. doi: 10.3389/fmolb.2021.705141 (PMC8290080; doi:10.3389/fmolb.2021.705141)
Supplement: Supplementary file 1 [file DataSheet1.zip › Supplementary Figure S2.PPTX]

## Slide 1
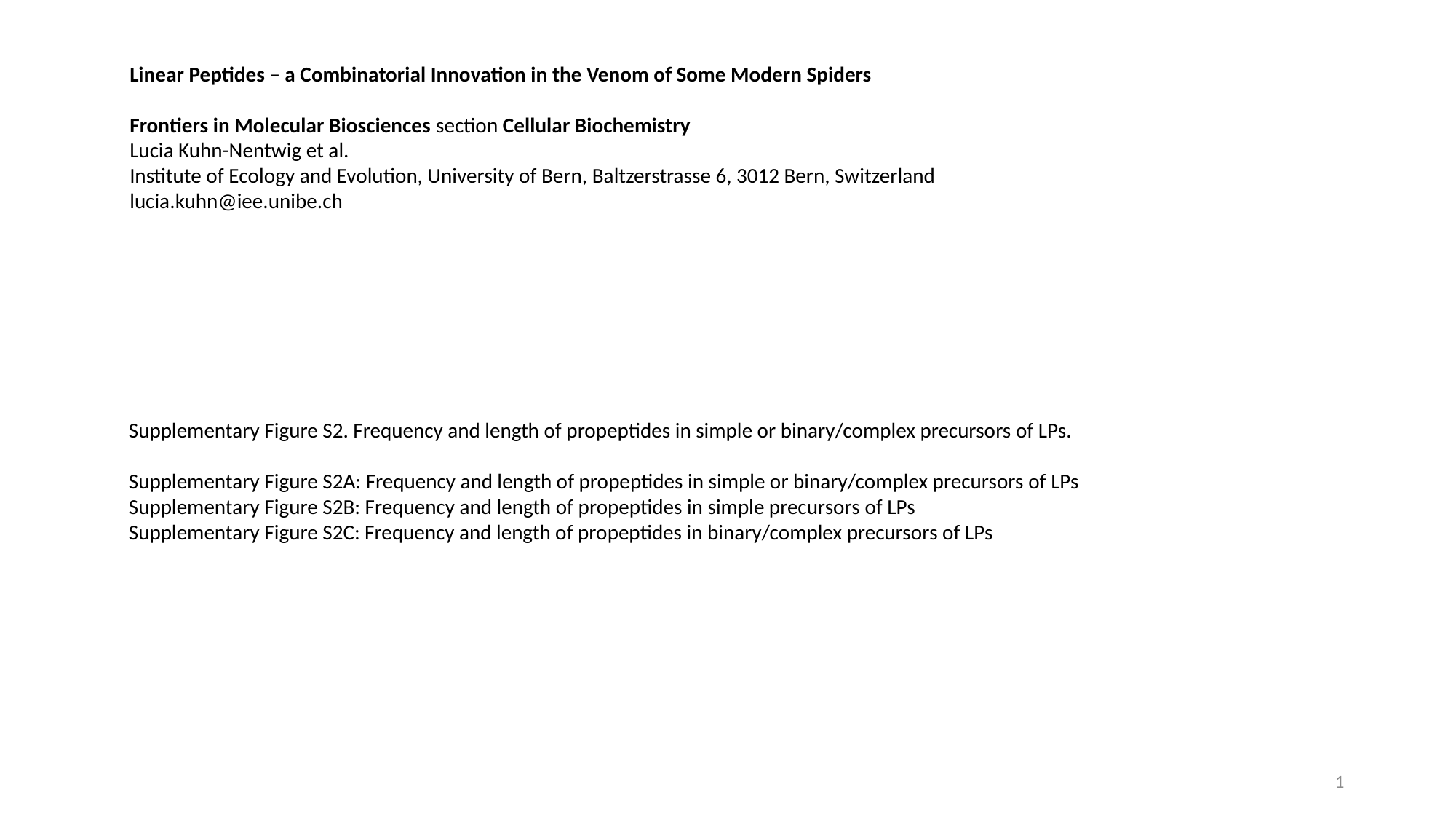

Linear Peptides – a Combinatorial Innovation in the Venom of Some Modern Spiders
Frontiers in Molecular Biosciences section Cellular Biochemistry
Lucia Kuhn-Nentwig et al.
Institute of Ecology and Evolution, University of Bern, Baltzerstrasse 6, 3012 Bern, Switzerland
lucia.kuhn@iee.unibe.ch
Supplementary Figure S2. Frequency and length of propeptides in simple or binary/complex precursors of LPs.
Supplementary Figure S2A: Frequency and length of propeptides in simple or binary/complex precursors of LPs
Supplementary Figure S2B: Frequency and length of propeptides in simple precursors of LPs
Supplementary Figure S2C: Frequency and length of propeptides in binary/complex precursors of LPs
1

## Slide 2
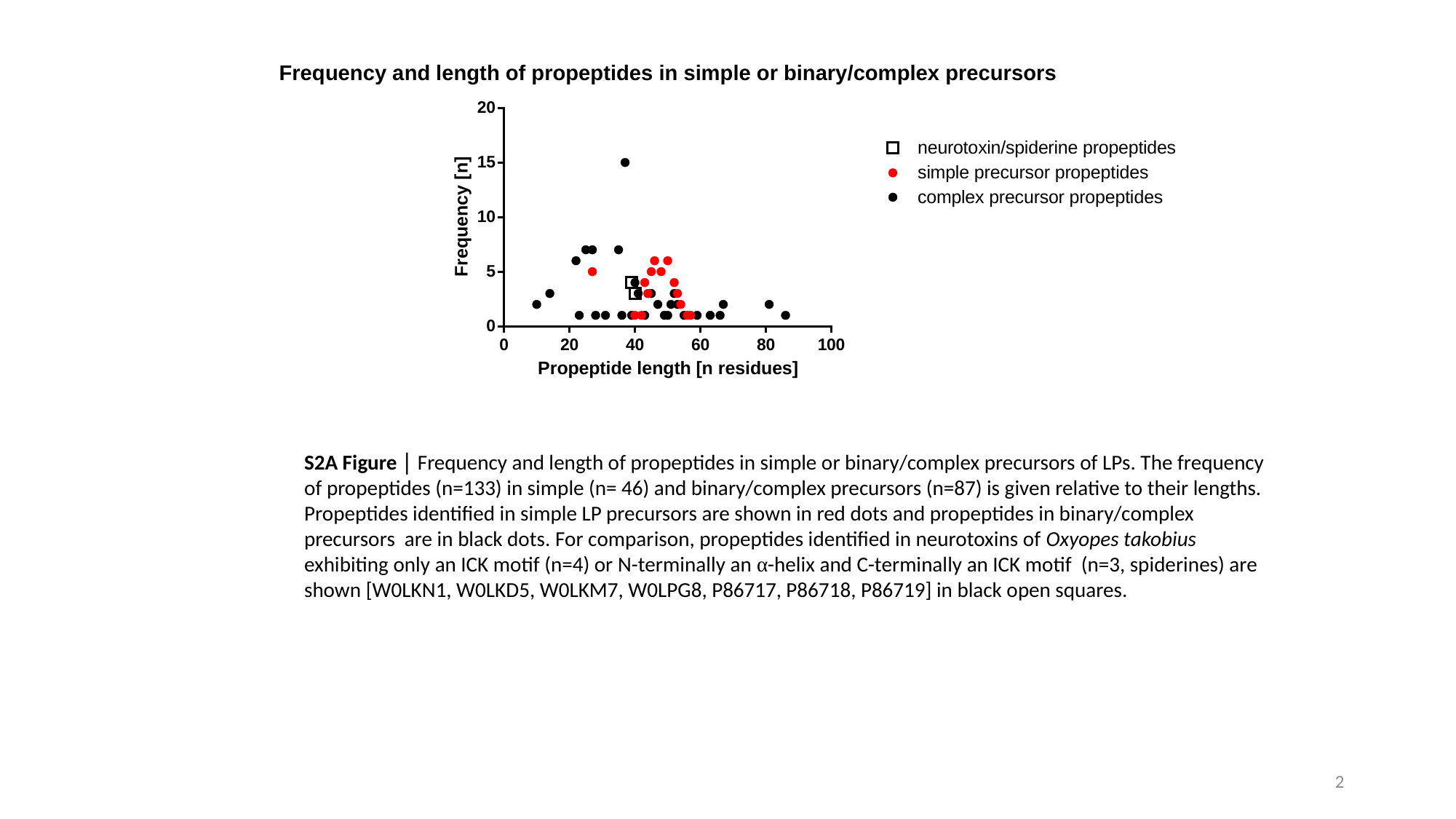

S2A Figure │ Frequency and length of propeptides in simple or binary/complex precursors of LPs. The frequency of propeptides (n=133) in simple (n= 46) and binary/complex precursors (n=87) is given relative to their lengths. Propeptides identified in simple LP precursors are shown in red dots and propeptides in binary/complex precursors are in black dots. For comparison, propeptides identified in neurotoxins of Oxyopes takobius exhibiting only an ICK motif (n=4) or N-terminally an α-helix and C-terminally an ICK motif (n=3, spiderines) are shown [W0LKN1, W0LKD5, W0LKM7, W0LPG8, P86717, P86718, P86719] in black open squares.
2

## Slide 3
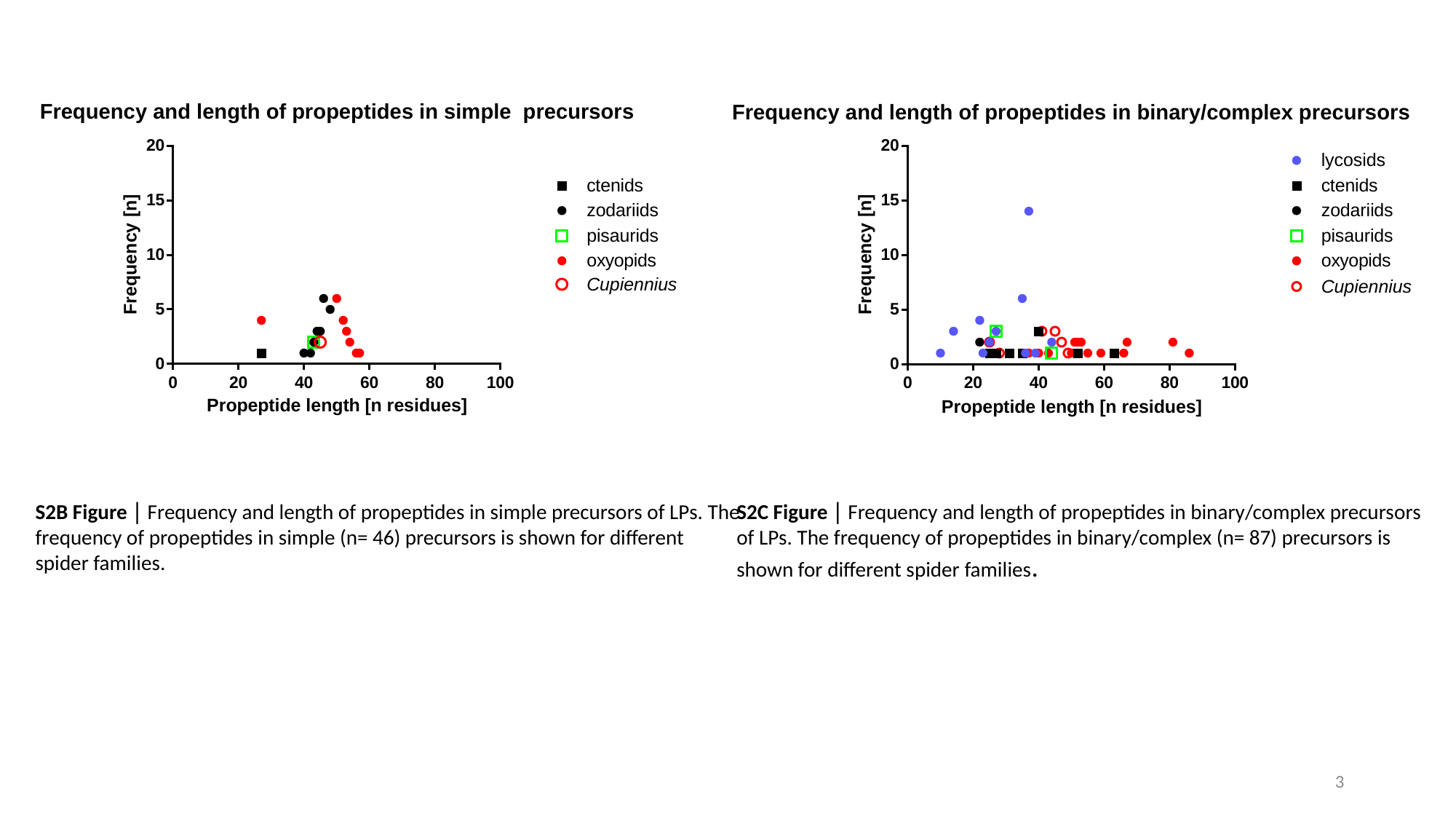

S2B Figure │ Frequency and length of propeptides in simple precursors of LPs. The frequency of propeptides in simple (n= 46) precursors is shown for different spider families.
S2C Figure │ Frequency and length of propeptides in binary/complex precursors of LPs. The frequency of propeptides in binary/complex (n= 87) precursors is shown for different spider families.
3
