# Supplementary material for: Linear Peptides—A Combinatorial Innovation in the Venom of Some Modern Spiders
Source: Front Mol Biosci. 2021 Jul 6;8:705141. doi: 10.3389/fmolb.2021.705141 (PMC8290080; doi:10.3389/fmolb.2021.705141)

**Linear Peptides – a Combinatorial Innovation in the Venom of Some Modern Spiders**

**Frontiers in Molecular Biosciences** section **Cellular Biochemistry**
Lucia Kuhn-Nentwig et al.
Institute of Ecology and Evolution, University of Bern, Baltzerstrasse 6, 3012 Bern, Switzerland
lucia.kuhn@iee.unibe.ch

Supplementary Figure S3. Overview on species specific linker motifs.

Supplementary Figure 3A: Species specific N-terminal iPQM motif of linkers
Supplementary Figure 3B: Species specific C-terminal PQM motif of linkers

# Species specific N-terminal iPQM motif of linkers

## Lycosids

*Alopecosa cuneata* N = 34
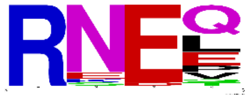


*Alopecosa marikovskyi* N = 10
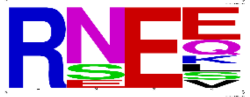


*Geolycosa vultuosa* N = 29
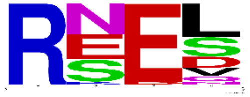


*Hogna radiata* (Spain) N = 32
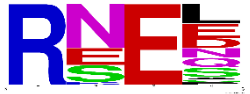


*Hogna radiata* (Italy) N = 29
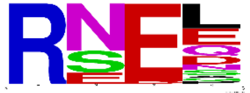


*Lycosa hispanica* N = 13
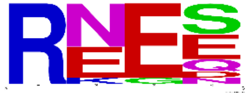


*Lycosa praegrandis* N = 11
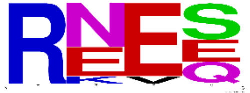


*Pardosa amentata* N = 23
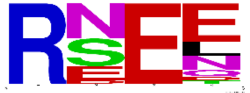


*Pardosa palustris* N = 52
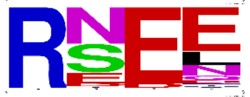


*Trochosa ruricola* N = 27
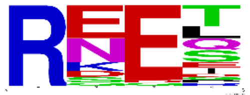


*Vesubia jugorum* N = 13
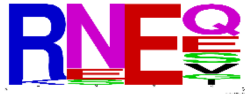


## Zodariids

*Lachesana tarabaevi* N = 5
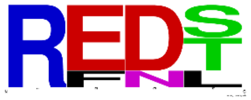


## *Pisaurids*

*Dolomedes fimbriatus* N = 1
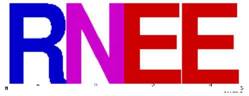


*Dolomedes okefinokensis* N = 2
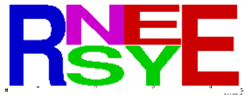


## *Cupiennius*

*Cupiennius getazi* N = 40
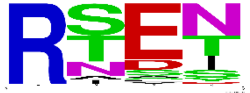


*Cupiennius salei* N = 91
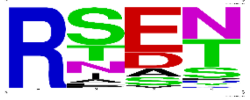


## Ctenids

*Macroctenus kingsleyi* N = 4
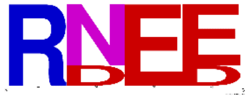


*Phoneutria fera* N = 6
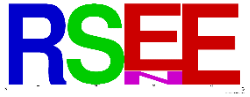


*Piloctenus haematostoma* N = 10
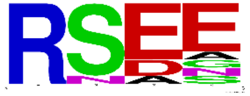


## Oxyopids

*Oxyopes heterophthalmus* N = 13
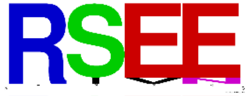


*Oxyopes lineatus* N = 17
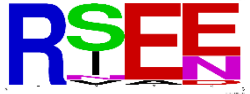


*Oxyopes takobius* N = 4
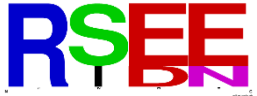


*Peucetia striata* N = 19
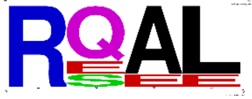

## Species specific C-terminal PQM motif of linkers

### Lycosids

*Alopecosa cuneata* N = 34
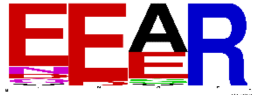

*Alopecosa marikovskyi* N = 10
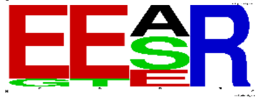

*Geolycosa vultuosa* N = 29
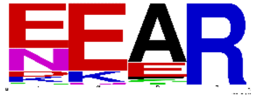

*Hogna radiata* (Spain) N = 32
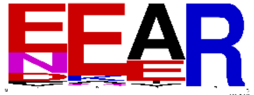

*Hogna radiata* (Italy) N = 29
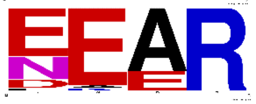

*Lycosa hispanica* N = 13
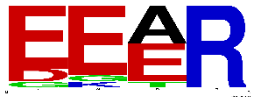

*Lycosa praegrandis* N = 11
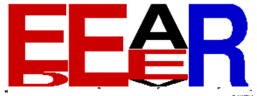

*Pardosa amentata* N = 23
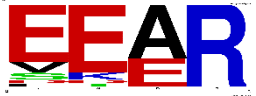

*Pardosa palustris* N = 52
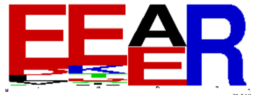

*Trochosa ruricola* N = 27
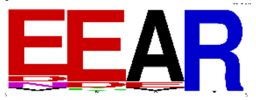

*Vesubia jugorum* N = 13
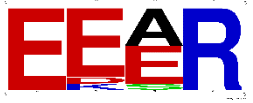

### Zodariids

*Lachesana tarabaevi* N = 5
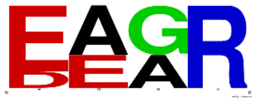

### *Pisaurids*

*Dolomedes fimbriatus* N = 1
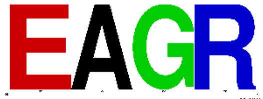

*Dolomedes okefinokensis* N = 2
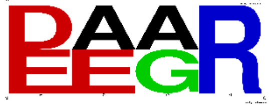

### *Cupiennius*

*Cupiennius getazi* N = 40
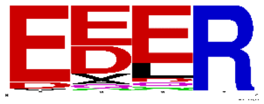

*Cupiennius salei* N = 91
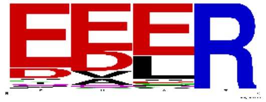

### Ctenids

*Macroctenus kingsleyi* N = 4
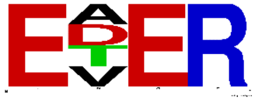

*Phoneutria fera* N = 6
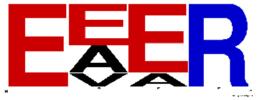

*Piloctenus haematostoma* N = 10
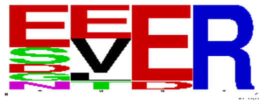

### Oxyopids

*Oxyopes heterophthalmus* N = 13
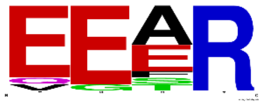

*Oxyopes lineatus* N = 17
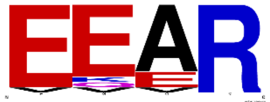

*Oxyopes takobius* N = 4
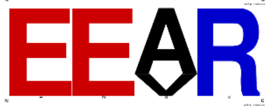

*Peucetia striata* N = 19
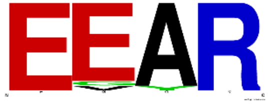

Supplement: Supplementary file 1 [file DataSheet1.zip › Supplementary Figure S3.PDF]
