# Supplementary material for: Linear Peptides—A Combinatorial Innovation in the Venom of Some Modern Spiders
Source: Front Mol Biosci. 2021 Jul 6;8:705141. doi: 10.3389/fmolb.2021.705141 (PMC8290080; doi:10.3389/fmolb.2021.705141)

Linear Peptides – a Combinatorial Innovation in the Venom of Some Modern Spiders

Frontiers in Molecular Biosciences section **Cellular Biochemistry**
Lucia Kuhn-Nentwig et al.
Institute of Ecology and Evolution, University of Bern, Baltzerstrasse 6, 3012 Bern, Switzerland
lucia.kuhn@iee.unibe.ch

Supplementary Figure S4. Overview on amino acid sequences of different peptide families identified in Lycosidae.

Supplementary Figure 4A: Amino acid sequences of alopecosins
Supplementary Figure 4B: Amino acid sequences of geolycosins
Supplementary Figure 4C: Amino acid sequences of hognins
Supplementary Figure 4D: Amino acid sequences of lycosins
Supplementary Figure 4E: Amino acid sequences of pardosins
Supplementary Figure 4F: Amino acid sequences of trochosins

Abbreviations:

ALOCU	*Alopecosa cuneata*
ALOMA	*Alopecosa marikovskyi*
GEOVU	*Geolycosa vultuosa*
HOGRS	*Hogna radiata* (Spain)
HOGRI	*Hogna radiata* (Italy)
LYCHI	*Lycosa hispanica*
LYCPR	*Lycosa praegrandis*
PARAM	*Pardosa amentata*
PARPA	*Pardosa palustris*
TRORU	*Trochosa ruricola*
VESJU	*Vesubia jugorum*

Alopecosin 1, 2, 3, 4, 5, 6, 7, 8 peptide families

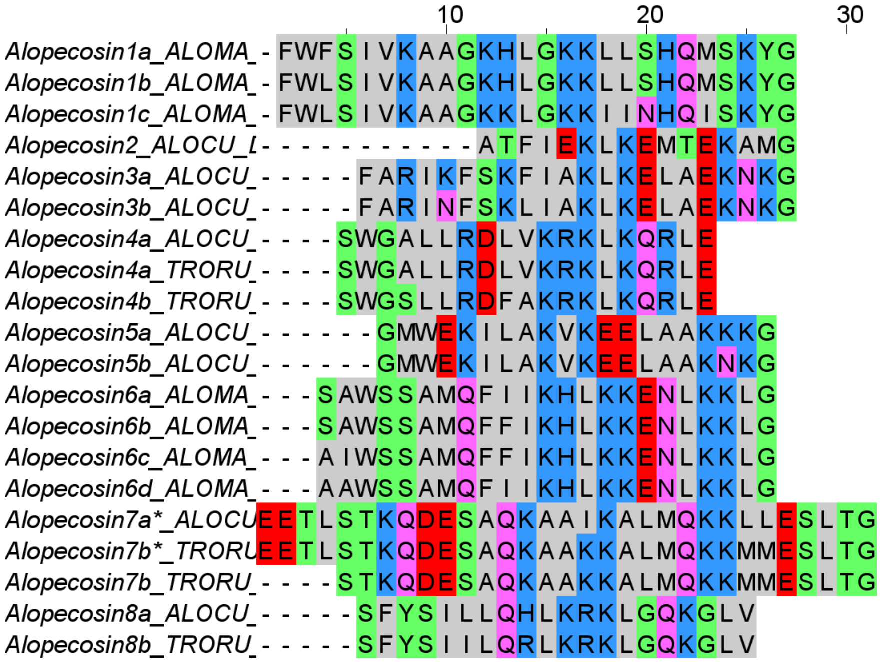

Geolycosin 1, 2, 3, 4 peptide families

```
                        10
Geolycosin1a_GEOVU_  -SLLGLLDVVKNTAGQTGLL
Geolycosin1b_GEOVU_  -SLLGLLDVVKKTVGQTGLL
Geolycosin1c_GEOVU_  -SLFGLLDVVKKTVGQTGLL
Geolycosin1d_GEOVU_  -SLLGVLDLVKSKVGQTGLL
Geolycosin1e_GEOVU_  -SLLGLLDIVKSKVGQTGLL
Geolycosin1f_GEOVU_  -ALFGLLDLVKSKVGQTGLL
Geolycosin1g_GEOVU_  -ALLGLLDLVKSKVGQTGLL
Geolycosin1h_GEOVU_  -SLLGLLDLVKNKVGQTGLL
Geolycosin1i_GEOVU_  -SLLGLLDLVKSKGGQPGLL
Geolycosin1j_HOGRI_  -SLLGLLDLVKNTVGQTGLL
Geolycosin1k_HOGRI_  -SLFGLLDLVKNTVGQTGLL
Geolycosin1k_HOGRS_  -SLFGLLDLVKNTVGQTGLL
Geolycosin1l_HOGRI_  -SLFGLLDLVKSKVGQTGLL
Geolycosin1l_HOGRS_  -SLFGLLDLVKSKVGQTGLL
Geolycosin1m_HOGRI_  -SLLGLLDLVKSKVGQTGLL
Geolycosin1m_HOGRS   -SLLGLLDLVKSKVGQTGLL
Geolycosin1n_HOGRS_  -SLLGLLDLVKSKIGQTGLL
Geolycosin1o_ALOCU_  -SLLGLLDFVKRKIGQTGLL
Geolycosin1o_TRORU_  -SLLGLLDFVKRKIGQTGLL
Geolycosin1p_ALOCU_  -SLLGLLDFVKRKVGQTGLL
Geolycosin1q_ALOCU_  -SLLGLLDFVKHKVGQTLLL
Geolycosin1r_GEOVU_  -SLRGLLDVVKNTVGQTGLL
Geolycosin1s_GEOVU_  -SLLGLLDVVKNTVGQTGLL
```

```
                             10        20
Geolycosin2a_GEOVU_  -SLLGLLDLVKNKVGQTVGLL
Geolycosin2b_GEOVU   SLLGGVLDVVKNTAGQTGLL
Geolycosin2c_GEOVU   SLLGGVLDVVKNTVGQAGLL
Geolycosin2d_GEOVU   SLLGGVLDVVKNTVGQTGLL
Geolycosin2e_GEOVU   SLLGGLLDVVKNTAGQTGLL
Geolycosin2f_GEOVU_  SLLGGLLDVVKNTVGQTGLL
Geolycosin2g_GEOVU   PLLGGVLDVVKNTVGQTGLL
Geolycosin2h_HOGRS_  -SLLGLLDHVTSKVGQTVGLL
Geolycosin2i_HOGRI_  -SLLGLLDHVLSKVGQTVGIL
Geolycosin2j_GEOVU_  SLLGGVLDVVKKTVGQTGLL
Geolycosin3a_GEOVU_  -SLLGLLDVVKNTAGQTGLLRKDVVSDNKA
Geolycosin3b_GEOVU   SLLGGVLDVVKNTAGQTGLLRKDVVSDNKA
Geolycosin4a_GEOVU_  -GRLRDFIKKLKAYIRKMKAKYSKATA
Geolycosin4b_GEOVU_  -GRLRDFINKLKAYIRKMKAKYSKATA
```

Hognin 1, 2, 3, 4, 5, 6, 7, 8 peptide families

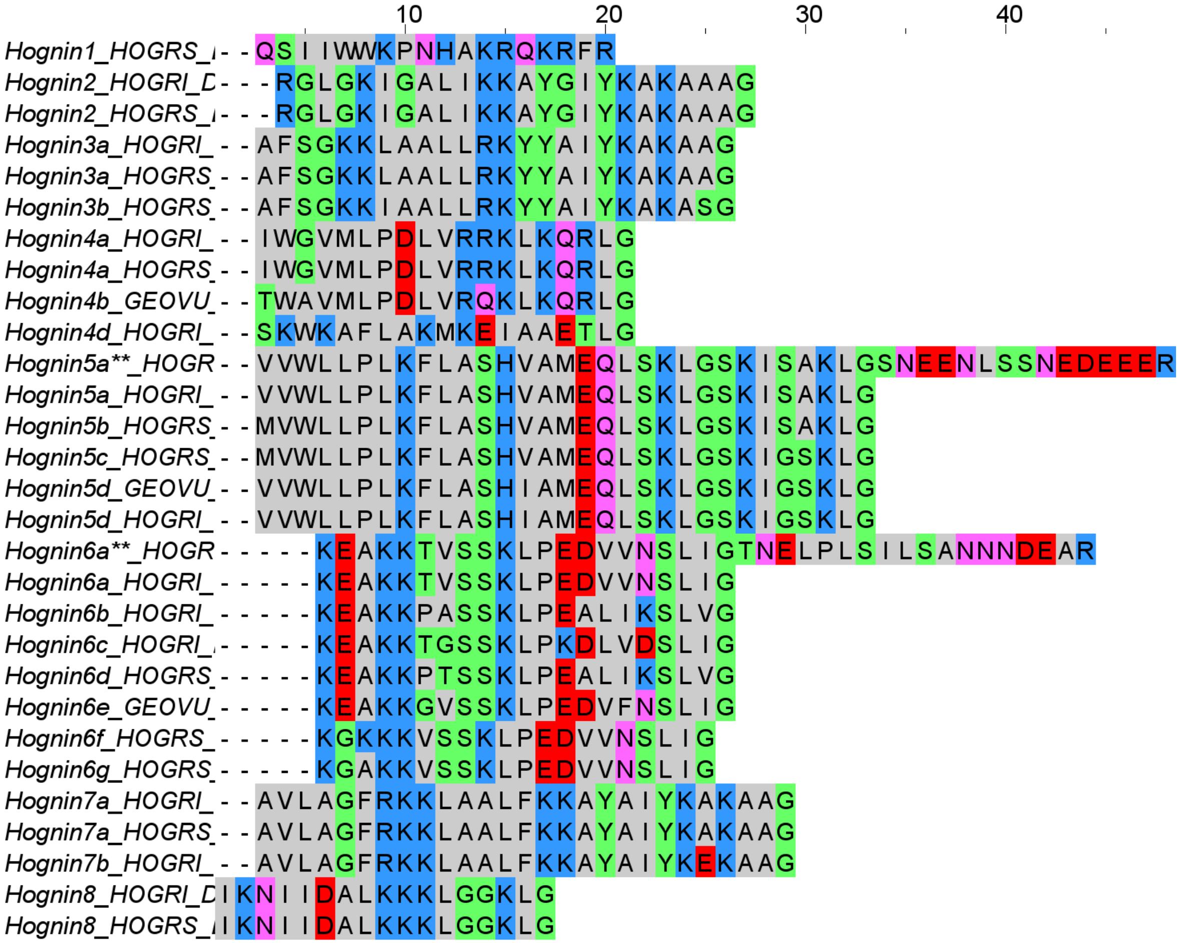

Lycosin 1, 2, 3, 4, 6, 7 peptide families

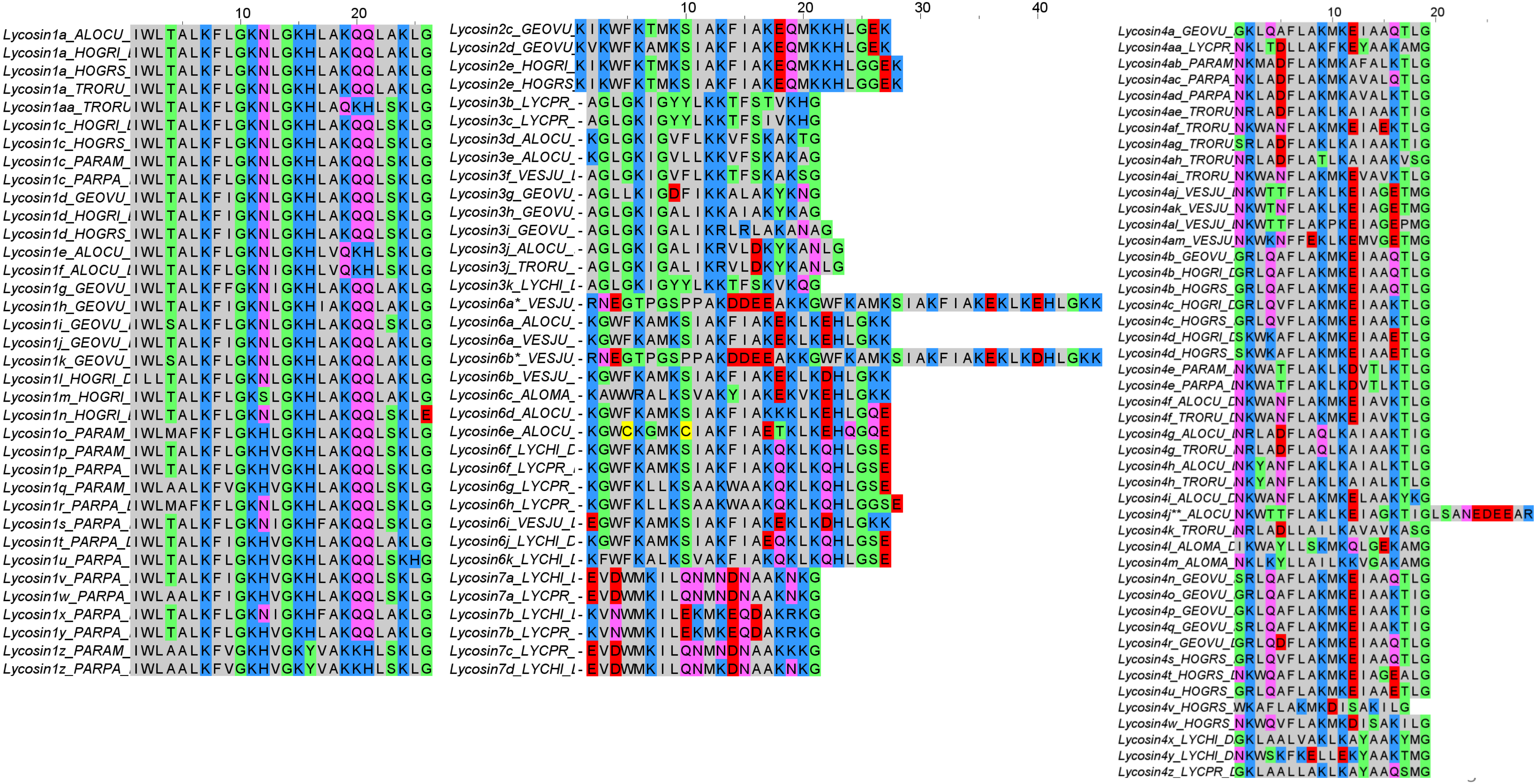

Lycosin 5, 8, 9 peptide families in public databases

Sequence	Alignment (positions 10, 20 marked)
Lycosin5a_GEOVU	VIWLPALKFLASHIAMEQLSKLG
Lycosin5a_HOGRI	VIWLPALKFLASHIAMEQLSKLG
Lycosin5a_HOGRS	VIWLPALKFLASHIAMEQLSKLG
Lycosin5a_PARAM	VIWLPALKFLASHIAMEQLSKLG
Lycosin5a_PARPA	VIWLPALKFLASHIAMEQLSKLG
Lycosin5b_ALOCU	IIWLPALKFLASHVVMEQLSKLG
Lycosin5b_TRORU	IIWLPALKFLASHVVMEQLSKLG
Lycosin5c_ALOCU	IIWLPVLKFLASHVAMEQLSKLG
Lycosin5c_PARAM	IIWLPVLKFLASHVAMEQLSKLG
Lycosin5c_PARPA	IIWLPVLKFLASHVAMEQLSKLG
Lycosin5d_PARAM	IIWLPALKFLASHIAMEQLSKLG
Lycosin5d_PARPA	IIWLPALKFLASHIAMEQLSKLG
Lycosin5d_TRORU	IIWLPALKFLASHIAMEQLSKLG
Lycosin5e_PARAM	IIWLPALKFLASHVAMEQLSKLG
Lycosin5e_PARPA	IIWLPALKFLASHVAMEQLSKLG
Lycosin5f_ALOCU	LIWLPALKFLASHIAMEQLSKLG
Lycosin5f_VESJU_l	LIWLPALKFLASHIAMEQLSKLG
Lycosin5g_ALOCU	LIWLPALKFLASHIAMEHISKLG
Lycosin5h_ALOCU	IIWLPALKFLASHIAMEHISKLG
Lycosin5i_ALOMA	IFWLPVAKFIASHIAMEQLSKLG
Lycosin5j_ALOMA	IIWLPLVKLIASHIAMEQLSKLG
Lycosin5k_LYCPR	IIWAPLLKFLATEVAMHHLSKMG
Lycosin5l_TRORU	LIWLPALKFLASHIAMEQISKLG
Lycosin5m_VESJU	VIWIPALKFLASHIAMEQLSKLG
Lycosin5n_LYCHI_l	LICIAALKFLSTEMVMHHLSKMG
Lycosin5o_LYCHI_l	LIWIPALKFLASEMVMHHLSKMG
Lycosin5p_LYCHI_l	LIWIPALKFLATELAMHHLSKMG
Lycosin5q_LYCHI_l	LIWIPALKFLATEVAMHHLSKMG
Lycosin5r_LYCHI_L	LFWIPALKFLATEVAMHHLSKMG

Sequence	Alignment (positions 10, 20 marked)
Lycosin8a_ALOCU	AAWLSAMKFLAKHLTKENLKKLG
Lycosin8a_VESJU	AAWLSAMKFLAKHLTKENLKKLG
Lycosin8b_ALOCU	AAWLSALKFLGKHFTKEQLKKLG
Lycosin8b_VESJU	AAWLSALKFLGKHFTKEQLKKLG
Lycosin8c_HOGRS	AAWLSAMKFLAKHLTKEQLKKLG
Lycosin8d_VESJU	AAWSSAMKFLAKYLTKENLKKLG
Lycosin8e_VESJU	AAWSSAMKFLAKYLTKENLKKFG
Lycosin8f_LYCPR	AAWLSAFKFLGKHFTKEQLKKYG
Lycosin8g_ALOMA	APVSAVIKFLAKYLNKENIKKFG
Lycosin8h_ALOMA	APVSAVIKFLAKYLNKENIKYLG
Lycosin8i_ALOMA	AAVSAVIKFLAKYLNKENIKKFG
Lycosin8j_ALOCU	AAFSAAMKFLAKYLTKENLMKLG
Lycosin8k_LYCPR	AAFISIRKILGKYFTKEQLKKYG
Lycosin8l_LYCHI_L	AAWLSALKHLVKHVTKEQFKKYG
Lycosin8o_VESJU	EARSSAMKFLAKYLTKENLKKLG
Lycosin8m_LYCHI	AAWLSALKHLVKHVTKEQLKKYG
Lycosin8n_LYCHI_l	AAWLSALKHLVKHVTKEHFKKYG
Lycosin9a_VESJU	-VWLTALKFLGKQLAKHQLSKLG
Lycosin9b_VESJU	-IWLTALKFLGKHLAKHQLSKLG
Lycosin9c_LYCPR	-IWGTVLKMFAKQVAKHQLSKMG
Lycosin9d_LYCPR	-IWGSVLKMFAKQVAKHQFSKMG
Lycosin9e_LYCPR	-IWGSVLKMFAKQVAKHQLSKMG
Lycosin9f_PARPA	-IWLMAFKFLGKHLAKQQLSKLG
Lycosin9g_ALOCU	-IWLTALKFLGKQLAKHQLSKLG
Lycosin9h_VESJU	-IWLTALKFLGKQRAKHQLSKRG
Lycosin9i_LYCHI_L	-IWLSLMKFAGKHLAKHQLSKMG
Lycosin9j_LYCHI_L	-IWWTAMKVLGKQLAKHQLSKMG
Lycosin9k_LYCHI_l	-MWWTAMKVLAKQLAKQHLSKMG
Lycosin9l_LYCHI_L	-IWFSLMKFAGKHLAKHQLSKMG
Lycosin9m_LYCHI_	-IWWTAMKFFGKQLAKHQLSKMG
Lycosin9n_LYCHI_l	-IWWTAMKFFGKQFAKHQLSKMG
Lycosin9o_LYCHI_l	-IWWTAVKFFGKQLAKHQLSKMG
Lycosin9p_LYCHI_l	-IWWTAVKYFGKQLAKLQLSKKG
Lycosin9q_LYCHI_l	-IWWTAMEFLGKQLAKHQLSKMG

Sequence	Alignment (positions 10, 20 marked)
sp|P61507|LYT1_HOGCA/1-25	IWLTALKFLGKHAAKHLAKQQLSKL
sp|C0HJU9|LYT1_LYCER/1-25	IWLTALKFLGKNLGKHLAKQQLAKL
sp|P61507|LYT1_HOGCA/1-25	IWLTALKFLGKHAAKHLAKQQLSKL
sp|P61508|LYT2_HOGCA/1-27	KIKWFKTMKSIAKFIAKEQMKKHLGGE
sp|P0C2U8|LYC3_LYCSI/1-26	KIKWFKTMKSLAKFLAKEQMKKHLGE
sp|P0C2U6|LYC1_LYCSI/1-18	GKLQAFLAKMKEIAAQTL
sp|P0C2U7|LYC2_LYCSI/1-18	GRLQAFLAKMKEIAAQTL
sp|P0C2U9|LYC34_LYCSI/1-19	AGIGKIGDFIKKAIAKYKN
sp|P0C2V0|LYC40_LYCSI/1-21	MIASHLAFEKLSKLGSKHTML

↑ Uniprot identifier

Pardosin 1, 2, 3, 4, 5, 6, 7, 8, 9, 10, 11, 12, 13, 14 peptide families

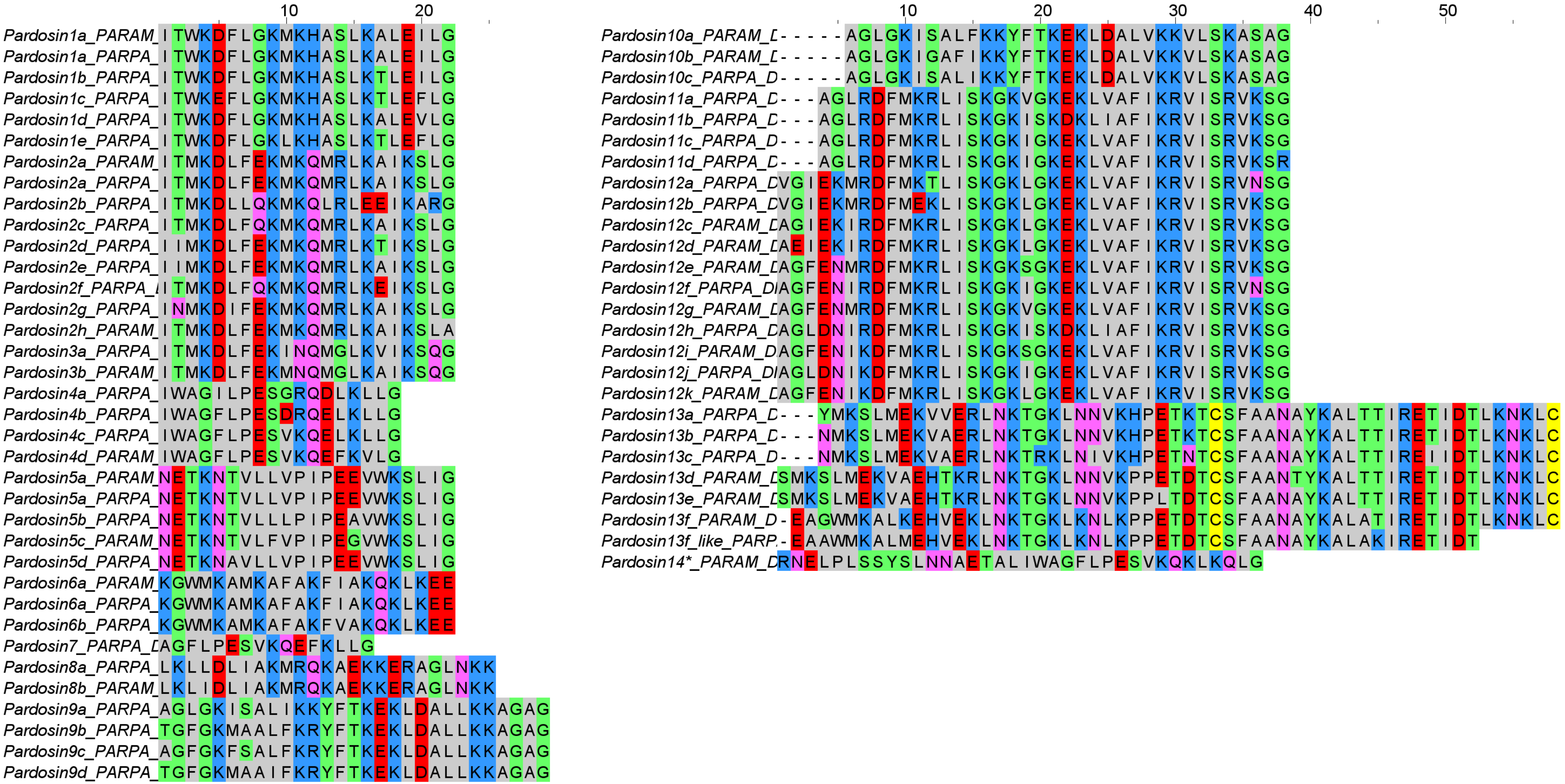

Trochosin 1, 2, 3, 4, 5 peptide families

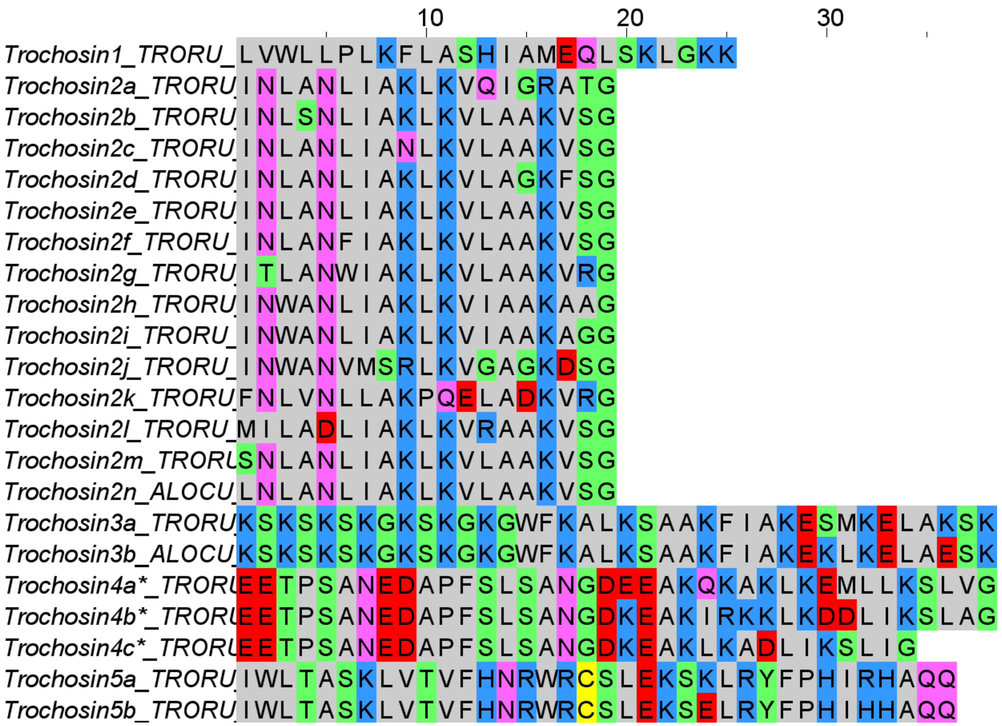

Supplement: Supplementary file 1 [file DataSheet1.zip › Supplementary Figure S4.PDF]
