# Supplementary material for: Linear Peptides—A Combinatorial Innovation in the Venom of Some Modern Spiders
Source: Front Mol Biosci. 2021 Jul 6;8:705141. doi: 10.3389/fmolb.2021.705141 (PMC8290080; doi:10.3389/fmolb.2021.705141)
Supplement: Supplementary file 1 [file DataSheet1.zip › Supplementary Table S6.PDF]

Supplementary Table S6 | Overview of LPs and peptide families identified in the transcriptomes of different lycosids.

Spider species	Peptide families (subfamilies)	Peptides	With other lycosids shared peptides	Fused peptides N-term* /C-term**	Cysteine containing peptides
Alopecosa cuneata	Alopecosin (2 – 5, 7, 8)<br>Geolycosin (1)<br>Lycosin (1, 3 – 6, 8, 9)<br>Trochosin (2, 3)	36	13	1*, 1**	1
Alopecosa marikovskyi	Alopecosin (1, 6)<br>Lycosin (4 – 6, 8)	15	0		
Geolycosa vultuosa	Geolycosin (1 – 4)<br>Hognin (4 – 6)<br>Lycosin (1, 2, 4, 5)	46	5		
Hogna radiata (Italy)	Geolycosin (1, 2)<br>Hognin (2 – 8)<br>Lycosin (1, 2, 4, 5)	27	18		
Hogna radiata (Spain)	Geolycosin (1, 2)<br>Hognin (1 – 8)<br>Lycosin (1, 2, 4, 5, 8)	33	17	2**	
Lycosa hispanica	Lycosin (3 – 9)	26	3		1
Lycosa praegrandis	Lycosin (3 – 9)	16	3		
Pardosa amentata	Lycosin (1, 4, 5)<br>Pardosin (1 – 6, 8, 10, 12 – 14)	32	12	1*	3
Pardosa palustris	Lycosin (1, 4, 5, 9)<br>Pardosin (1 – 13)	60	12		4
Trochosa ruricola	Alopecosin (4, 7, 8)<br>Geolycosin (1)<br>Lycosin (1, 3, 4, 5)<br>Trochosin (1 – 5)	41	9	4*	2
Vesubia jugorum	Lycosin (3 – 6, 8, 9)	20	4	2*	
Total		352	96	11	11
